# When can we detect lianas from space? Toward a mechanistic understanding of liana‐infested forest optics

**DOI:** 10.1002/ecy.70082

**Published:** 2025-04-27

**Authors:** Marco D. Visser, Matteo Detto, Félicien Meunier, Jin Wu, Jane R. Foster, David C. Marvin, Peter M. van Bodegom, Boris Bongalov, Matheus Henrique Nunes, David Coomes, Hans Verbeeck, J. Antonio Guzmán Q, Arturo Sanchez‐Azofeifa, Chris J. Chandler, Geertje M. F. van der Heijden, Doreen S. Boyd, Giles M. Foody, Mark E. J. Cutler, Eben N. Broadbent, Shawn P. Serbin, Stefan Schnitzer, M. Elizabeth Rodríguez‐Ronderos, Frank Sterck, José A. Medina‐Vega, Stephen W. Pacala

**Affiliations:** ^1^ Department of Ecology and Evolutionary Biology Princeton University Princeton New Jersey USA; ^2^ Institute of Environmental Sciences Leiden University Leiden the Netherlands; ^3^ Computational and Applied Vegetation Ecology, Department of Environment Ghent University Ghent Belgium; ^4^ Department of Earth & Environment Boston University Boston Massachusetts USA; ^5^ Laboratory for Space Research University of Hong Kong Hong Kong; ^6^ Rubenstein School of Environment & Natural Resources University of Vermont Burlington Vermont USA; ^7^ Southern Research Station US Forest Service Knoxville Tennessee USA; ^8^ Salo Sciences, Inc. San Francisco California USA; ^9^ Department of Plant Sciences, Forest Ecology & Conservation Group University of Cambridge Cambridge UK; ^10^ Department of Geographical Sciences University of Maryland College Park Maryland USA; ^11^ Department of Plant Sciences The Conservation Research Institute, University of Cambridge Cambridge UK; ^12^ Centre for Earth Observation Sciences (CEOS), Earth & Atmospheric Sciences Department University of Alberta Edmonton Alberta Canada; ^13^ School of Geography University of Nottingham University Park Nottingham UK; ^14^ School of Social Sciences University of Dundee Dundee UK; ^15^ School of Forest Resources and Conservation University of Florida Gainesville Florida USA; ^16^ Terrestrial Ecosystem Science & Technology Group, Environmental Sciences Department Brookhaven National Laboratory Upton New York USA; ^17^ Department of Biological Sciences Marquette University Milwaukee Wisconsin USA; ^18^ Department of Geography National University of Singapore Singapore Singapore; ^19^ Forest Ecology and Management Group Wageningen University Wageningen the Netherlands; ^20^ Forest Global Earth Observatory, Smithsonian Tropical Research Institute Washington DC USA

**Keywords:** albedo, biodiversity, leaf angles, leaf optics, leaf traits, radiative transfer models

## Abstract

Lianas, woody vines acting as structural parasites of trees, have profound effects on the composition and structure of tropical forests, impacting tree growth, mortality, and forest succession. Remote sensing could offer a powerful tool for quantifying the scale of liana infestation, provided the availability of robust detection methods. We analyze the consistency and global geographic specificity of spectral signals—reflectance across wavelengths—from liana‐infested tree crowns and forest stands, examining the underlying mechanisms of these signals. We compiled a uniquely comprehensive database, including leaf reflectance spectra from 5424 leaves, fine‐scale airborne reflectance data from 999 liana‐infested canopies, and coarse‐scale satellite reflectance data covering 775 ha of liana‐infested forest stands. To unravel the mechanisms of the liana spectral signal, we applied mechanistic radiative transfer models across scales, establishing a synthesis of the relative importance of different mechanisms, which we corroborate with field data on liana leaf chemistry and canopy structure. We find a consistent liana spectral signal at canopy and stand scales across globally distributed sites. This signature mainly arises at the canopy level due to direct effects of more horizontal leaf angles, resulting in a larger projected leaf area, and indirect effects from increased light scattering in the near and short‐wave infrared regions, linked to lianas' less costly leaf construction compared with trees on average. The existence of a consistent global spectral signal for lianas suggests that large‐scale quantification of liana infestation is feasible. However, because the traits responsible for the liana canopy‐reflectance signal are not exclusive to lianas, accurate large‐scale detection requires rigorously validated remote sensing methods. Our models highlight challenges in automated detection, such as potential misidentification due to leaf phenology, tree life history, topography, and climate, especially where the scale of liana infestation is less than a single remote sensing pixel. The observed cross‐site patterns also prompt ecological questions about lianas' adaptive similarities in optical traits across environments, indicating possible convergent evolution due to shared constraints on leaf biochemical and structural traits.

## INTRODUCTION

Lianas are woody vines that act as structural parasites of trees and have a strong influence on forest dynamics (Visser, Wright, et al., 2018), decreasing tree growth and survival (Ingwell et al., 2010) and arresting forest succession (Tymen et al., 2016). Lianas are increasing in abundance across the tropics (Rueda‐Trujillo et al., 2024), raising concerns about the potential negative impact on carbon sequestration (Durán et al., 2013; van der Heijden et al., 2015). However, the spatial extent of this increase is uncertain, and the causes remain mostly unknown (De Deurwaerder et al., 2024; Schnitzer, 2015). Remote sensing could provide a powerful tool to quantify this trend on a broad range of spatiotemporal scales and elucidate the climatic and bio‐geographical factors contributing to liana proliferation (van der Heijden et al., 2022). Yet, as of today, we do not know whether a globally distinct liana spectral signal exists that could make such research possible.

Ecologists have utilized various remote sensing platforms and methods to detect lianas, including multispectral and hyperspectral sensors on drones, aircraft, and satellites (Chandler et al., 2021; Foster et al., 2008; Kaçamak et al., 2022; Li et al., 2018; Marvin et al., 2016; Tymen et al., 2016; Waite et al., 2019). These studies suggest that lianas have distinct detectable spectral signals at the canopy and stand scales. However, differentiating lianas and trees at the leaf level might depend on forest type, with detectable differences observed in dry forests but not in wet forests (Asner & Martin, 2012; Guzmán Q. et al., 2018; Sánchez‐Azofeifa, Castro, et al., 2009). This suggests that climatic factors influence the chemical and structural properties of liana leaves compared to their hosts (Asner & Martin, 2012; Medina‐Vega, Bongers, Schnitzer, & Sterck, 2021; Werden et al., 2018).

The influence of climate indicates exceptions and contextual dependencies rather than a distinct global liana spectral signal. This raises doubts about the applicability of automated classifiers for quantifying liana abundances across large spatiotemporal domains. Data‐driven automated classifiers can capture complex relationships but may struggle to generalize beyond the training data, particularly when critical relationships are missing (Féret et al., 2019; Meyer & Pebesma, 2022; Willard et al., 2022). Thus, the major challenge in robustly applying automated classification techniques lies in collecting training data representative of all conditions, which may not be feasible across large spatial domains with different climates or floristic compositions. Collecting representative training data in tropical forests is especially daunting due to the high biodiversity. For instance, many leaf and canopy traits are known to vary systematically with life history and leaf phenology among plant groups, but differences can be small compared with the large variability across interspecific, intraspecific, phenotypic, and ontogenetic levels of natural vegetation (Castro‐Esau et al., 2004; Detto & Xu, 2020; Kitajima et al., 2005; Sánchez‐Azofeifa, Castro, et al., 2009; Werden et al., 2018; Wu et al., 2018; Zhang et al., 2006).

At a more fundamental level, pure data‐driven automated classifiers do not explain the underlying reasons responsible for a plant spectral signal. Alternatively, a process‐based modeling approach can offer fundamental mechanistic insights essential for robust remote detection of biological phenomena. A causal mechanistic understanding enables predicting how the signal changes across regions and spatial scales (Wu et al., 2018), and this aids in the development of algorithms that adapt across varied conditions, reducing misclassification errors and increasing effectiveness despite environmental changes A basic understanding of underlying processes facilitates optimized study and sensor design at wavelength and spatial scale that accurately target phenomena of interest (Verstraete et al., 2015), or cross‐platform integration (e.g., Rao et al., 2020), while identifying potential sources of error by clarifying confounding factors from biophysical phenomena (Soudani & François, 2014), or species with similar spectral properties (Tropek et al., 2014). Currently, a thorough ground‐validated mechanistic understanding of the liana signal across spatial scales from leaf to canopy is lacking, which limits our ability to confidently monitor the large‐scale impacts of lianas on forest ecosystems.

In this study, we compile a uniquely comprehensive dataset that spans the visible, near infrared (NIR), and short‐wave infrared (SWIR) spectrum (400–2500 nm) across different biogeographic regions and spatial scales for hundreds of species. Our goal is to pioneer an understanding of the mechanisms behind the spectral signal of liana leaves and canopies, compared with host trees, by linking species traits to reflectance through mechanistic radiative transfer models (RTMs) and corroborating these findings with field data. Our objectives include: (1) determining if a geographically consistent liana spectral signal exists across globally dispersed sites, detectable at various spatial scales and across different remote sensing tools; (2) identifying traits that create a liana signal via radiative transfer modeling; (3) validating results with independent field data; (4) quantifying trait importance; and (5) evaluating the effectiveness of spectral indices and classifiers in detecting lianas at different scales using simulated spectra.

## METHODS

### Overview

**FIGURE 1:**
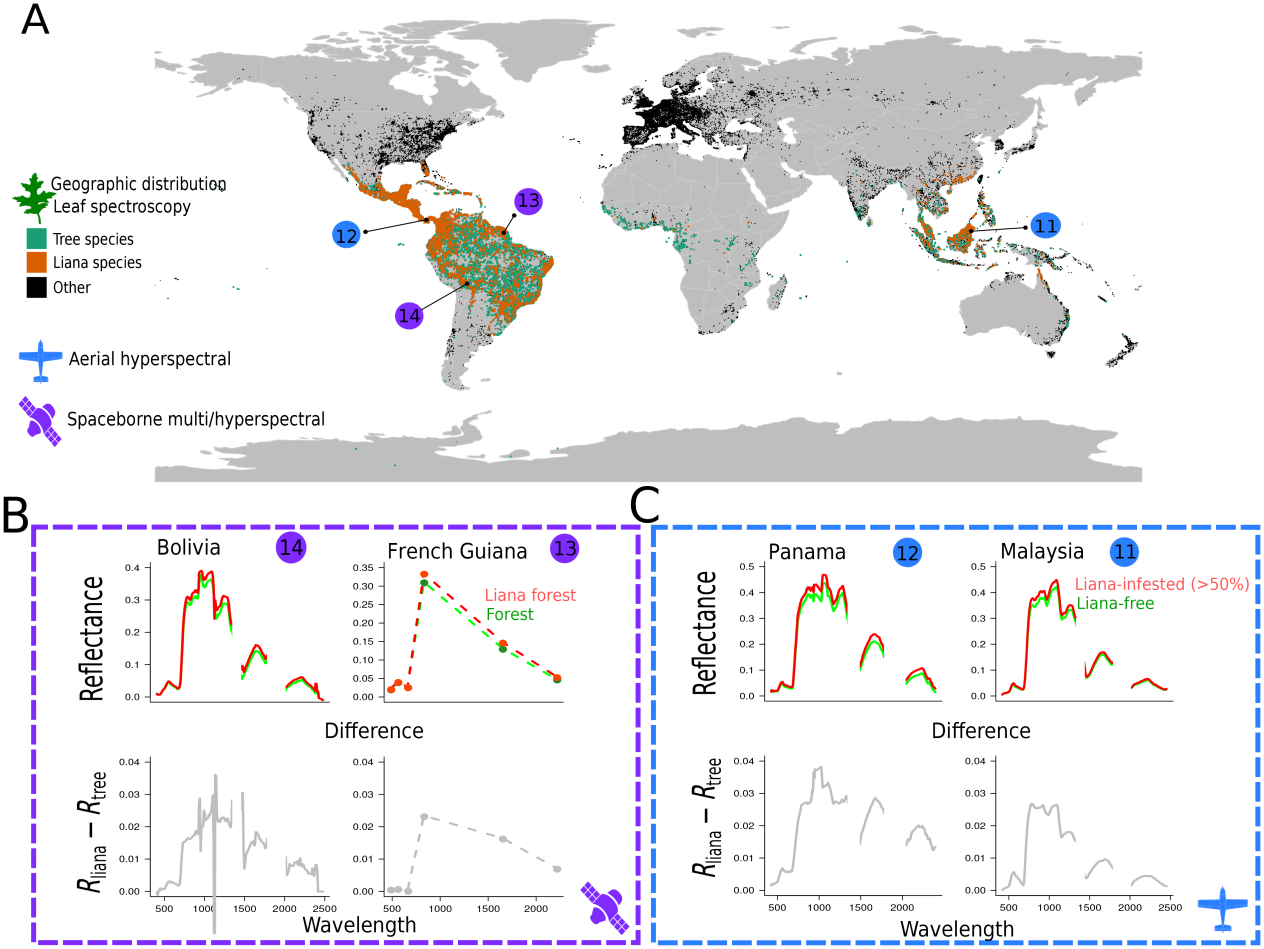
Datasets used in the study to compare the tropical liana and tree spectral signals. Panel A shows the geographic distribution of plants species in the leaf spectroscopy dataset, including the focal tropical liana (orange) and tree species (green). Note that no sampling was conducted in Africa, but three species are common to both the Americas and Africa: *Parinari excelsa*, *Symphonia globulifera*, and the liana *Byttneria catalpifolia*. The locations and remote sensing platform types of canopy scale data are given in blue and purple with reference numbers corresponding to Appendix S1: Table S1. Panels B and C display liana spectral signals from airborne and spaceborne sensors at focal sites. Top rows show surface reflectance signals of lightly infested forests (*R*_tree_) and heavily infested forests (*R*_liana_) at each location. Bottom rows show average differences between *R*_lianas_ and *R*_trees_. Illustrations by Marco D. Visser.

To test all objectives, we compiled liana reflectance data in the 400–2500‐nm range from multiple sites, sensors, and scales (Figure 1, Appendix S1: Table S1). To test *geographic and scale signal consistency* (objective 1), we first compiled and analyzed a comprehensive dataset on liana reflectance at the leaf level and compared it with other plant growth forms. Then, we analyzed datasets of studies that successfully distinguished between tropical lianas and host trees at canopy and stand scales. Next, to conduct *trait identification and validation* (objectives 2 and 3), we inversely fitted mechanistic RTMs to estimate differences in leaf biophysical, leaf chemical, and canopy architectural properties (here after “traits”) between lianas and host trees and validated them with ground measurements. We assessed the *relative importance* of each trait (objective 4) in generating the observed liana reflectance distribution (mean and variation) through iterative model experiments. Finally, using forward simulations of the fitted models, we evaluated the potential of different platforms to discriminate lianas and trees under various scenarios (objective 5: *platform potential*).

### Study sites, species and data

#### Spectral datasets

The spectral data (Figure 1, Appendix S1: Table S1) span three distinct spatial scales and integrate data from 17 independent sources spanning multiple sites and scales. To conserve space and improve readability, details on data, field, and laboratory protocols are provided in the supplemental information (Appendix S1: Section S1.2). Further details are given in the original published sources.

*Leaf scale* (<<1‐m^2^ scale) data encompassed 11 published reflectance spectra datasets covering 5424 leaves of 720 species. Our focus is primarily on tropical species, with 648 tropical tree species and 66 liana species. To place liana leaf reflectance globally, we also included data on 72 crops, shrubs, and herbaceous species in the SI, as all groups could potentially lead to confounding in large classification studies (as shown in e.g., Tropek et al., 2014). In all datasets, reflectance was measured in the 300–2500‐nm range (<2‐nm step) with laboratory or field spectroradiometers (Appendix S1: Table S1 and Section S1.2). Unique species counts were obtained by cross‐referencing names with the GBIF database and tallying unique GBIF keys. Geographic distributions were also sourced from GBIF and cleaned following Zizka et al. (2019).

*Canopy scale* (1–4‐m^2^ scale) data were obtained from two published airborne hyperspectral campaigns in Panama and Malaysia, covering reflectance from 999 tree crowns with varying levels of ground‐truthed liana infestation. Further details on the crown‐scale data, sites, and instruments can be found in Appendix S1, Chandler et al. (2021) and Marvin et al. (2016).

*Stand scale* (900‐m^2^ scale) data used satellite imagery from previous studies to contrast liana‐infested and liana‐sparse forest patches in Bolivia and French Guiana (Foster et al., 2008; Tymen et al., 2016). In both cases, we obtained the original images used, which included a 30‐m resolution Landsat Thematic Mapper (L1TP) image (French Guiana) and hyperspectral Hyperion imagery from NASA's EO‐1 satellite (Bolivia). Reflectance data span over 775 ha of forest (40.5 in French Guiana, 734.76 in Bolivia) and was classified as low liana density forest and adjacent heavily infested and successionally arrested stands (Foster et al., 2008; Tymen et al., 2016). In French Guiana, the liana forest was delineated using lidar, aerial photography, and field campaigns, while in Bolivia, it was contrasted with random forest patches using high‐resolution videography and field surveys. Both images were georeferenced and surface reflectance corrected. The Hyperion image was corrected for the smile effect. Detailed information on the methods and sites can be found in Tymen et al. (2016), Foster et al. (2008), and Appendix S1: Section S1.

### Leaf and canopy traits

To achieve our trait identification and validation objectives (2 and 3), we combined published and newly collected field data on leaf and canopy traits, including data on lianas and host tree species from Panama and Malaysia.

#### Leaf biochemical and biophysical trait data

We obtained measurements of leaf Chlorophyll (*C*_ab_, in micrograms per square centimeter), Carotenoids (*C*_ar_, in micrograms per square centimeter), water content (*C*_w_, in grams per square centimeter), leaf thickness (in micrometers) and leaf mass area (*C*_m_, in grams per square centimeter). Trait measurements followed typical protocols, where pigment contents were determined from leaf discs punched from fresh leaves, and water content was measured by recording fresh mass before drying. A subset of the data included matched leaf spectral reflectance and physically measured traits for the same leaves (Table 1). The paired leaf data were used to benchmark the inversion of leaf RTMs (see Appendix S1). Average trait values for lianas and trees were also available for dataset 3 in Sánchez‐Azofeifa, Castro, et al. (2009). Finer details on sampling, extraction, and quantification can be found in Appendix S1.

**TABLE 1:** Model parameters and associated canopy and leaf traits.

Scale and trait	Parameter	Units	Description	Validation dataset
Leaf				
Leaf structural parameter	*N*	Unitless	Average no. air‐cell wall interfaces (where light passes between the refractive index for water to air) within the mesophyll. Expected to correlate strongly with leaf thickness.	1, 2, 3
Chlorophyll *a* and *b* content per unit leaf area	*C*_ab_	μg/cm^2^	Primary photosynthetic pigment.	1, 2, 3
Carotenoid content per unit leaf area	*C*_ar_	μg/cm^2^	Secondary photosynthetic pigment.	1, 2, 3
Water mass per unit leaf area	*C*_w_	g/cm^2^	Known as the “equivalent water thickness,” the hypothetical thickness of water distributed over the area of a remotely sensed pixel because the parameter's units reduce to cm when “divided” by the density of pure water (1 g/cm^3^).	1, 2, 3, 11
Leaf dry matter per area	*C*_m_	g/cm^2^	Oven‐dried mass over fresh area.	1, 2, 3, 11
Anthocyanin content	*C*_an_	μg/cm^2^	Protective flavonoids shielding photostems from excess light and herbivory.	Estimated (calibration parameter)
Brown pigments content	*C*_br_	Arbitrary	Colored residue of degraded pigments during senescence or after stress.	Estimated (calibration parameter)
Canopy				
Leaf Area Index	LAI	Unitless	Projected area of all leaves over a unit of land (m^2^/m^2^).	13
Leaf inclination angle	Ω	Degrees	The average angular orientation of the leaf normal relative to the zenith.	17
Fraction secondary particles	*f*_2_	Unitless	Proportion of secondary (tree) leaf particles in canopy as a fraction of the total LAI, with 1 − *f*_2_ giving the fraction of primary leaves (lianas).	16
Canopy dissociation factor	*D*	Unitless	Mixing of primary (liana) and secondary (tree) particles, ranging between 0 and 1, with 0 a perfectly homogeneous mixture and 1 all primary leaves in the top layer.	Fixed (set to 1)
Hotspot	κ	Unitless	Particle coarseness, defined as the ratio of the mean (projected) particle size over the width of the canopy layer (*b*/*H*).	Estimated
Soil moisture	Ω	Unitless	Soil layer reflectance is influenced by soil moisture (0–1).	Estimated
Level of infestation	*L*	Unitless	Liana infestation index (0–1) known from data.	Fixed
Stand				
Vertical crown cover fraction	Cv	Unitless	Fraction ground area covered by foliage at nadir view (Carea/A).	Fixed (set to 1)
Crown shape	ζ	Unitless	Crown diameter over tree height (Cd/H).	Fixed
Sensor				
Solar zenith angle	θ_s_	Degrees	Complement of sun elevation in degrees from horizon (90° − sun elevation).	Fixed
Observer zenith angle	θ_o_	Degrees	Sensor zenith angle (datasets often nadir corrected).	Fixed
Sun‐sensor relative azimuth angle	ψ	Degrees	Relative azimuth angle, or difference between sun and observer azimuth angles.	Fixed
All				
Error	σ	Unitless	Residual error on predicted reflectance ρ̂.	Estimated

#### Plant area index (PAI)

The contribution of liana leaves to PAI in infested canopies was estimated using a plant canopy analyzer (LAI2000, LI‐COR) (dataset 16). The estimation capitalized on a liana removal experiment in central Panama, where the contribution of lianas to PAI was estimated from the difference between control and removal plots (Appendix S1: Section S1.2). Details regarding the LI‐COR measurements are given in Appendix S1 and Rodríguez‐Ronderos et al. (2016), while van der Heijden et al. (2015) describe the removal experiment. Note that we use PAI to refer to empirical measurements, and LAI to refer to the PROSAIL model parameter as per convention.

#### Leaf angles

Leaf angle distribution was measured for canopies of 18 tree species and 19 liana species in Panama (dataset 17). Samples were taken in the canopies of trees and lianas using leveled‐digital photography (Pisek et al., 2011; Ryu et al., 2010). Images were taken at different locations, including canopy cranes, telecommunication towers, eddy‐covariance flux towers, and canopy gaps. We measured sun and shaded leaves for trees, but only sun leaves for lianas, as the vast majority of liana leaves are in the sunexposed canopy (Avalos et al., 1999; Medina‐Vega, Bongers, Poorter, et al., 2021). Leaf angles were defined as the angular orientation of the leaf surface normal relative to the zenith (Ross, 1981) and measured from digital pictures using imageJ (Rueden et al., 2017). Whenever the leaves were compound, the angle from the petiole to the furthest leaf tip relative to the zenith was taken as the leaf angle. A total of 1540 individual leaves (646 tree and 894 liana leaves) were measured (Appendix S1: Figure S1; Detto et al., 2015).

### Radiative transfer models (RTMs)

**FIGURE 2:**
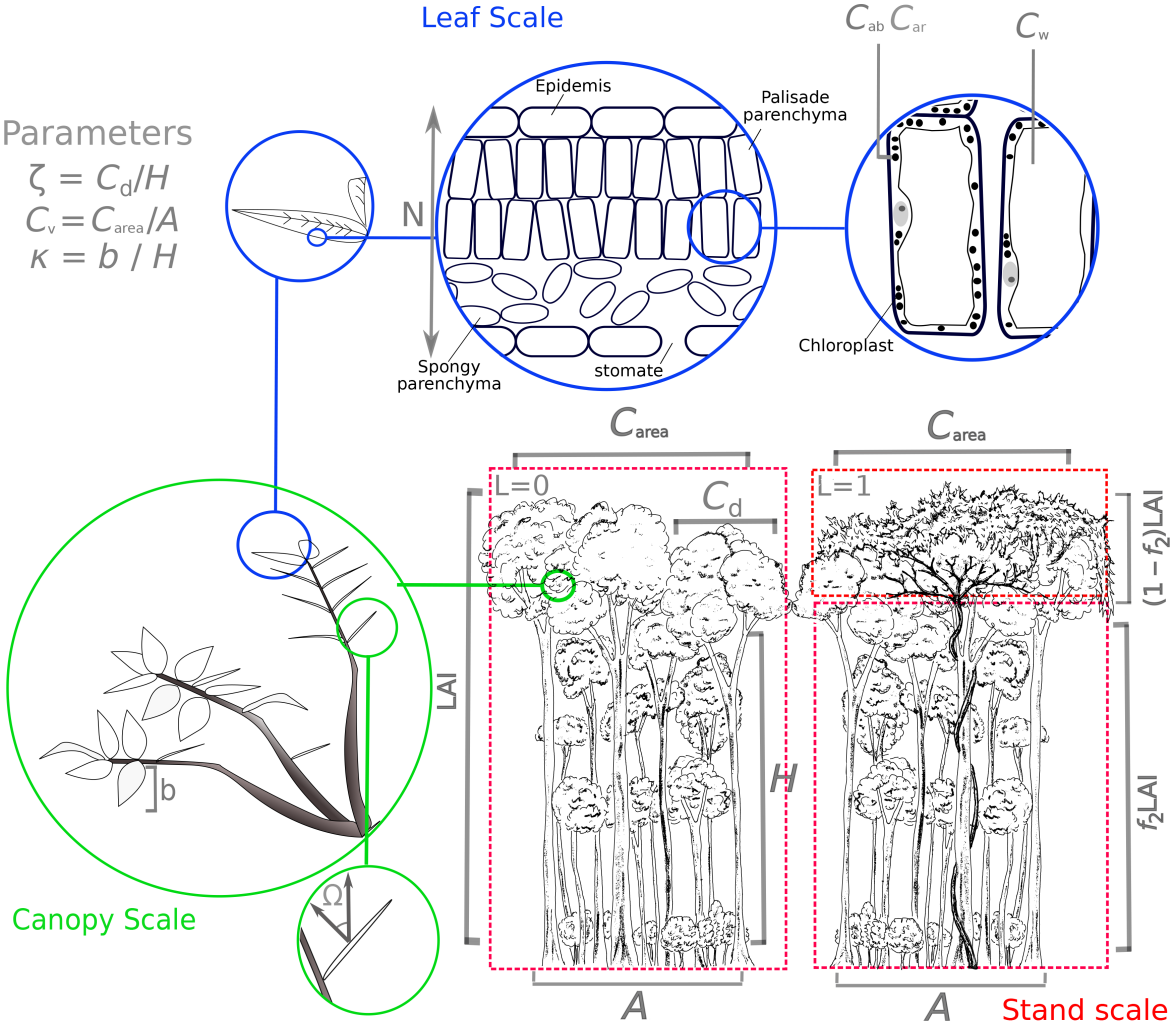
Illustration of the coupled radiative transfer model, merging PROSPECT and FOURSAIL2 to study light behavior in vegetation canopies. PROSPECT addresses leaf radiative transfer, using semitransparent stacked plates to represent leaf absorption (α), transmittance (τ), and reflectance (ρ). It employs the generalized plate model to determine wavelength‐dependent transmissivity (*k*(λ)), based on empirical absorption coefficients and leaf biochemical concentrations (see Table 1). FOURSAIL2 simulates bi‐directional reflectance at the canopy level, using differential equations for direct and hemispherical radiance. It splits the canopy into two vertical and infinite horizontal layers with randomly distributed leaves. The model integrates leaf properties (α, ρ, τ) from PROSPECT and allows for different leaf types in the canopy, either layered or mixed, using the canopy dislocation parameter (*D*). In our study, *D* was set to 1, placing liana leaves predominantly above host leaves. Canopy layer optical thickness is defined by sensor‐sun geometry (θ_s_, θ_o_, ψ), affecting radiance flux length and the angular projection of leaf area. This projection relies on mean leaf angle (Ω_1_, Ω_2_) and leaf area index (LAI), split between top (liana; 1 − *f*_2_), and bottom (tree; *f*_2_) layers. The model includes a soil layer with variable reflectance and a moisture parameter (ω) and considers the hotspot effect, where equal observer and solar zenith angles increase reflectance. The hotspots intensity is calculated as canopy coarseness (leaf width to canopy height ratio, κ = *b*/*H*), and the model also accounts for crown cover (Cv) and shape (ζ). However, we only study dense tropical forests, so Cv was set to 1, simplifying the model by negating ζs impact on reflectance. Illustrations by Marco D. Visser.

To address objectives 1–5, we utilized RTMs from the PROSPECT and SAIL families, which have been extensively validated and applied in reflectance modeling studies (e.g., Shiklomanov et al., 2016; Wu et al., 2018; Zhang et al., 2005). All models were programmed in R, optimized for speed following Visser et al. (2015), and refactored in C++ using Rcpp (Eddelbuettel & François, 2011). Model code is available as the open‐source R package ccrtm (coupled chain RTMs; version 0.1.6; Visser, 2020). We provide a short overview in Figure 2, Table 1, and below, as these models are exhaustively described elsewhere (see e.g., Jacquemoud & Ustin, 2019).

#### Leaf model

PROSPECT models leaf absorption, transmittance, and reflectance by simulating radiative transfer through semitransparent stacked plates representing leaf cell‐air interfaces (see Figure 2). Within the PROSPECT family of models, we evaluated PROSPECT 5, 5b, and D (Féret et al., 2017) for their precision in predicting independently measured trait data. We fitted each model to reflectance data for individual leaves and selected the model (5, 5b, or D) that closely reproduced the validation data. Traits, including water mass per unit area, leaf mass area, chlorophyll content, and carotenoids, were validated using paired reflectance and trait data from datasets 1, 2, and 11. Leaf thickness (in micrometers) indirectly validated the leaf structure parameter (*N*) in datasets 1, 2, 3, and 11, as thicker leaves typically have more air‐cell interfaces. Some parameters, such as brown pigments (*C*_br_) and anthocyanin content (*C*_an_), lacked validation data and were treated as calibration parameters: they were only kept if their inclusion improved both the quality of fit and the validation of other parameters (see also Féret et al., 2017). Details on the fit procedure are given below, and all parameters and their units are given in Table 1. A preliminary analysis among leaf models showed PROSPECTD performed the best with regard to reducing errors in both the fit and validation datasets, and therefore further analyses focus solely on PROSPECTD inversions.

#### Canopy model

We adapted 4SAIL2, which can represent vertically separated vegetation types while maintaining analytical tractability (Verhoef & Bach, 2003, 2007; Zhang et al., 2005). Lianas typically form a canopy on top of their host crown (Avalos et al., 1999; Stevens, 1987), with empirical studies finding the vast majority of liana leaves in the upper‐most leaf layers (Medina‐Vega, Bongers, Schnitzer, & Sterck, 2021). This vertical mixing of vegetation types has nonlinear impacts on reflectance, rendering linear mixing inappropriate (Asner, 1998). The 4SAIL2 model simulates the reflectance of two‐component canopies with distinct vertically separated layers, accounting for differences in leaf optics, optical thickness (LAI), and leaf inclination angles (Braswell et al., 1996). We provide a short description specific to the liana‐host tree model implemented here in Figure 2.

#### Coupled chain models

The PROSPECT and SAIL families of models can be coupled together in a chain, typically known as PROSAIL, where the outputs from the former are used as inputs for the latter. Here, we coupled PROSPECTD with 4SAIL2 to simulate canopy and stand‐scale reflectance in liana‐infested forests. The full PROSAIL2 model, in its most complete form, is given by:

(1a) $${\hat{\tau }}_{i},{\hat{\rho }}_{i}=\text{PROSPECT}\left(\lambda ,N_{i},C_{\mathrm{ab},i},C_{\mathrm{ar},i},C_{\text{an},i},C_{\mathrm{br},i},C_{w,i},C_{m,i}\right)\;i=1,2,$$

(1b) $${\hat{\rho }}_{d},{\hat{\rho }}_{s}=4\text{SAIL}2\left(\lambda ,{\hat{\tau }}_{1},{\hat{\rho }}_{1},{\hat{\tau }}_{2},{\hat{\rho }}_{2},\mathrm{LAI},L\;\times f_{2},\Omega_{1},\Omega_{2},D,\kappa ,C_{v},\zeta ,\theta_{s},\theta_{o},\psi \right),$$

(1c) $$\hat{\rho }=S\left(\lambda ,\theta_{s}\right){\hat{\rho }}_{d}+\left(1-S\left(\lambda ,\theta_{s}\right)\right){\hat{\rho }}_{s},$$

here, ${\hat{\tau }}_{i}$ and ${\hat{\rho }}_{i}$ are the predicted leaf transmittance and reflectance (i=1 for lianas, 2 for trees) at wavelength λ, while ${\hat{\rho }}_{s}$ and ${\hat{\rho }}_{d}$ represent bi‐directional (direct) and hemispherical directional reflectance (diffuse). The parameters $f_{2}$ and L divide the total leaf area (LAI) between the top and bottom layers, with L being the infestation intensity index (ranging between 0 and 1). The optical thickness of the top liana layer is assumed to be proportional to the liana infestation intensity ($L\times \left(1-f_{2}\right)$). This results in a maximum optical thickness of $\left(1-f_{2}\right)$ for the top layer when L=1, and the canopy reverts to a single layer with only tree leaves when the infestation is zero (L=0). The fraction of LAI assigned to lianas is then LAI × $L\;\left(1-f_{2}\right)$. The final predicted reflectance of the target pixel $\hat{\rho }$ depends on the fraction of diffuse light, $S\left(\lambda ,\theta_{s}\right)$, at wavelength λ and solar zenith angle $\left(\theta_{s}\right)$ (following Danner et al., 2019; François et al., 2002). Parameters detail and units are given in Table 1 and Figure 2.

### Inverse modeling of the liana signal

Spectral inverse modeling aims to estimate physical parameter values consistent with observed reflectance data (objective 2). We employed a Bayesian framework for spectral inverse modeling (Shiklomanov et al., 2016), where coupled chain RTM‐models are particularly useful, as models fitted at one scale can inform models at another scale. We first fit the PROSPECTD model to all 5424 individual leaf‐reflectance spectroscopy data, decomposing variation among groupings using a hierarchical modeling approach:

(2) $$T_{\mathrm{ijk}}\sim \beta_{0}+\beta_{i}+\beta_{\mathrm{jk}}+\beta_{k}+\varepsilon ,$$

where $T_{\mathrm{ijk}}$ represents one of the RTM model parameters in Equation (1a), fit for growth form *i* (liana or tree) from species *j* nested in study *k*. We included an effect of study ($\beta_{k}$) to account for variation due to factors associated with specific methodologies, such as the usage of different spectroradiometers (Meireles et al., 2020). All β parameters were assumed to be normally distributed with uninformative hyperpriors. We utilized leaf‐level means and variances to inform priors for leaf parameters at canopy and stand scales, assuming them to be normally distributed. The informative priors for both lianas and trees largely overlapped, with their means falling within one standard deviation of each other in every instance. All other parameters had uniform flat priors (see Appendix S1: Section S2 for details). For models fit at all scales, we sampled the joint posterior distribution of each RTM with Monte Carlo Markov Chain (MCMC) methods using the differential evolution adaptive metropolis algorithm, implemented in the “BayesianTools” R package (Hartig et al., 2019). In the inversion procedure we excluded the multispectral data from French Guiana (dataset 13) because parameter uncertainty is sensitive to spectral resolution (Shiklomanov et al., 2016). Therefore, we focus on hyperspectral data (datasets 12, 13, and 15), and included each sensor's spectral response functions in the fitting procedure. Finer details on model inversion are given in Appendix S1: Section S2. We conducted several model robustness checks, including simulations to determine parameter identifiability and the effect of measurement error (Appendix S1: Section S3).

### Spectral differences and the detection of the liana signal

Kullback–Leibler divergence (KLD) is a measure from information theory that quantifies the difference between probability distributions, commonly used for optimization in machine learning as a measure of how well classifiers can distinguish features (Kosheleva & Kreinovich, 2017). KLD is used here to explore differences in spectral distributions (reflectance, transmission, absorption) between plant groups (*signal consistency, obj 1*), the impact of traits on spectral distributions (*trait relative importance, obj. 4*), and the theoretical ability to distinguish lianas from trees (*platform potential*, *obj. 5*). At the leaf level, we used KLD to compare different plant groups, with tropical liana leaves as the reference (0). KLD is interpreted as the information lost when encoding the liana spectral distribution (0) with another plant group's distribution (*k*). KLD was defined as:

(3) $$d_{k0}\left(u_{k}|u_{0}\right)=\int_{400}^{2500}\int_{0}^{1}u_{k}\left(\rho ,\lambda \right)\log\frac{u_{k}\left(\rho ,\lambda \right)}{u_{0}\left(\rho ,\lambda \right)}d\lambda d\rho ,$$

where $u_{k}$ (ρ,λ) is the probability of observing a reflectance value ρ at wavelength λ, and $u_{0}$ (ρ,λ) is a reference distribution. Note, that we refer to a “spectral distribution” as the distribution of either reflectance, transmittance or absorption values across all wavelengths. Assuming normality, any spectral distribution can be estimated with the wavelength specific mean and standard deviation, $\mu \left(\lambda \right)$ and $\sigma \left(\lambda \right)$ respectively. In this case KLD (Equation 3) reduces to:

(4) $$d_{k0}\left(u_{k}|u_{0}\right)=\int_{400}^{2500}\;\log\left(\;\frac{\sigma {\left(\lambda \right)}_{0}}{\sigma {\left(\lambda \right)}_{k}}+\frac{\sigma {\left(\lambda \right)}_{0}^{2}+{\left(\mu {\left(\lambda \right)}_{k}-\mu {\left(\lambda \right)}_{0}\right)}^{2}}{2\sigma {\left(\lambda \right)}_{0}^{2}}-\frac{1}{2}\right)d\lambda .$$

The smaller the KLD value, the more comparable the groups are spectrally, which decreases the likelihood of robust classification (Kosheleva & Kreinovich, 2017). The expected KLD between two identically distributed signals with equal means and variances is, by definition, zero.

#### Model sensitivity experiment

KLD quantifies the loss or gain of information due to individual traits by comparing modeled spectral distributions that include or exclude one or more parameters. This provides a metric of individual traits' importance (objective 4). KLD was calculated with predicted reflectance $\hat{\rho }\left(\lambda \right)$ as the mean and variance estimated from the residual error from the inverse fits (σ, Appendix S1: Section S2). Starting with a model parameterized with mean tree leaf and canopy traits as the focal distribution $u_{k}$($\hat{\rho }$,λ), we iteratively changed single parameters to liana‐specific values and calculated the change in KLD using the liana spectral distribution as the reference distribution $u_{0}$($\hat{\rho }$,λ). This determines the parameters with the greatest information loss or gain toward the liana spectral distribution. To understand influential parameters at different wavelengths, we also evaluated the KLD at each wavelength separately and assessed combinations of two parameters to identify interactions that most strongly recreated the liana pattern.

#### The theoretical ability of classifiers to detect the liana signal

In theory, sensors and spectral indices that sample at bands maximizing KLD between trees and lianas optimize discernibility between these groups. Hence, sensor sampling regions of maximum KLD are expected to provide optimal information for modeling and classification (Wang et al., 2015). Therefore, we compare how well the spectral response functions of several common multispectral remote sensing platforms maximize KLD. Given the strong spatial clustering of lianas at different scales (Ledo & Schnitzer, 2014) and their varying infestation patterns from (1) incidental crown infestation (Marvin et al., 2016) to (2) arrested succession (Schnitzer et al., 2000) and (3) liana forests (Tymen et al., 2016), we investigate how the spatial aggregation scale interacts with sensor spatial resolution in determining the expected KLD. We simulated 1000 × 1000 pixel hyperspectral image scenes with various scales and autocorrelation degrees in liana infestation for scenarios (1–3). We started with the finest scale (1 m^2^) as pure signals and then recalculated the expected KLD between feature (liana) and non‐feature (liana‐free) areas, gradually reducing spatial resolution from 1 × 1 m to 250 × 250 m via resampling, repeating this 50 times per scenario. Each scenario maintained a roughly equal number of liana‐infested and noninfested pixels. The sensors modeled included Landsat TM, Worldview 2's WV110 camera, and Hyperion.

Note that we focus solely on the aspects of sensor choice readily controllable by the user. We explicitly ignore atmospheric effects that decrease the signal‐to‐noise ratio of most sensors (Landgrebe & Makaret, 1986). Consequently, the results should be considered a “best‐case scenario.”

## RESULTS

### Objective 1: Does a globally consistent liana signal exist at any scale?

**FIGURE 3:**
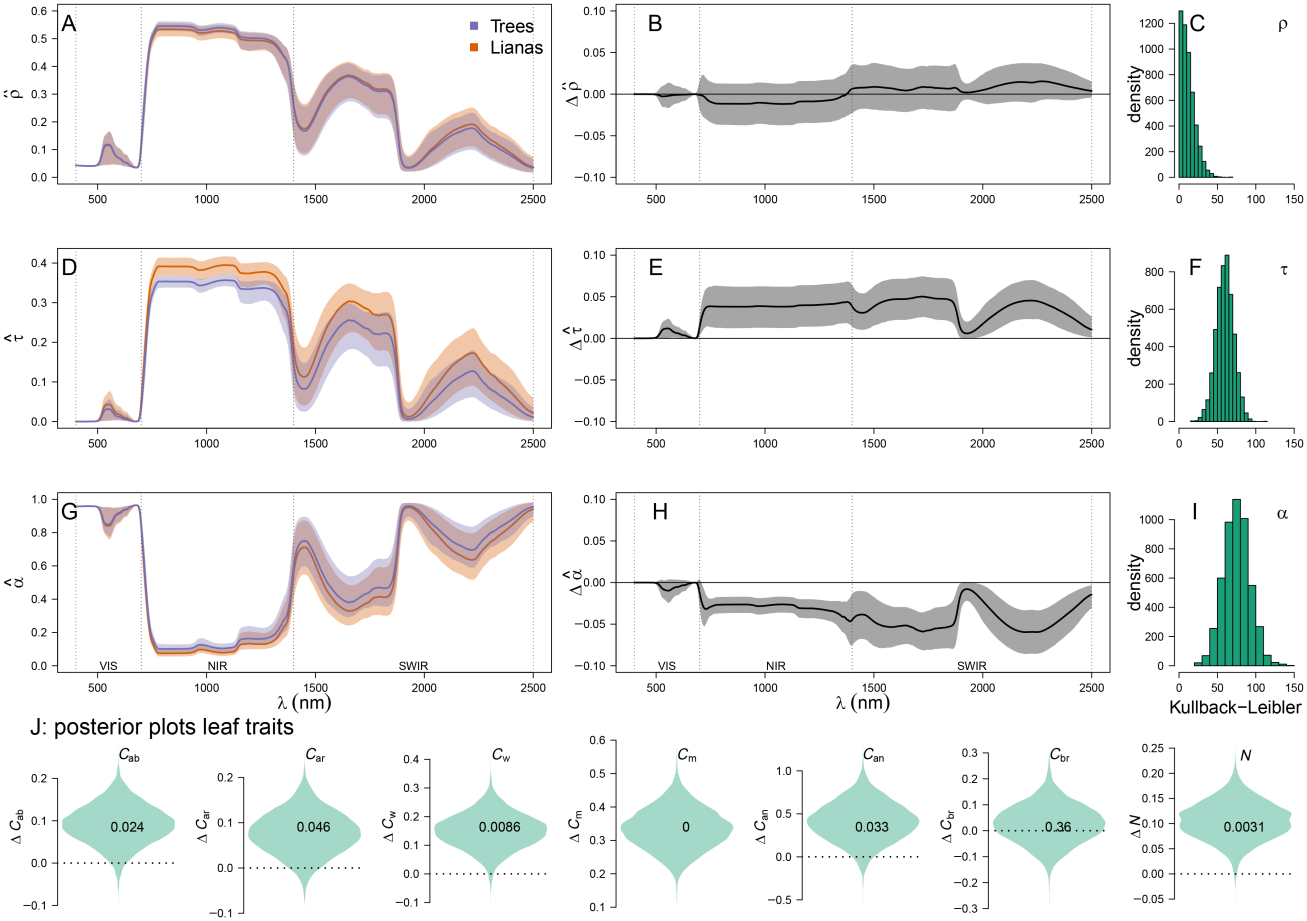
Spectral properties of liana and tree leaves in the solar spectrum (i.e., the region of peak solar irradiance spanning 400–2500 nm). Panels A to I show the predicted posterior distributions of reflectance $\left(\hat{\rho }\right)$, transmission ($\hat{\tau }$) and absorption ($\hat{\alpha }$) of liana and tree leaves (A, D, G) from PROSPECTD inversions on leaf reflectance data, and the difference between the posterior distributions (B, E, H)—here solid lines depict means and the shaded envelop shows 95% credible intervals. Kullback–Leiber Divergence (KLD) quantifies how the overall spectral probability distribution of trees diverges from liana for reflectance (*C*), transmission (*F*), and absorption (*I*)—with zero indicating an identical distribution. The violin plots in panel J, show differences between trees and lianas in predicted posterior distributions of leaf traits. From right to left; chlorophyll content (*C*_ab_), carotenoids (*C*_ar_), water mass per unit area (*C*_w_), leaf mass area (*C*_m_), anthocyanin content (*C*_an_), brown pigments (*C*_br_), and leaf structure (*N*). Fractions within each violin plot indicate the fraction of posterior samples that overlap with zero (i.e., no difference).

The distribution of liana leaf‐scale reflectance, measured from 718 leaves from 66 species, exhibited significant overlap with trees, shrubs, crops, and grasses (Appendix S1: Figure S2). This finding was further supported by inversely fit PROSPECT leaf models (Figure 3A–C). Kullback–Leibler divergence (KLD) analyses on spectral distributions and predicted posterior distributions confirmed the indistinguishable nature of tree and liana leaf reflectance across all wavelengths (Figure 3). In contrast to leaf reflectance, however, predicted posterior distributions of leaf transmission and absorption demonstrated substantial differences, particularly in the NIR and SWIR regions (Figure 3D–I). Liana leaves had higher transmission and lower absorption throughout the evaluated spectrum, leading to pronounced distributional divergence (Figure 3F,I).

Liana‐infested canopies showed consistently higher reflectance than liana‐free canopies at both canopy and stand scales across various sites, platforms, and wavelengths (upper panels, Figure 1B,C). When comparing the difference between liana and tree reflectance (lower panels, Figure 1B,C), (*R*_lianas_ − *R*_trees_) had an average 89% correlation between sites (range 74%–99%) when matched to Landsat 5TM bands (used in French Guiana). For hyperspectral images at overlapping wavelengths, the mean correlation for reflectance difference was 96% (range 95%–98%). These results reveal a distinct and detectable spectral signal for liana reflectance at canopy and stand scales, stable across globally dispersed sites, instrumental noise, and sensor differences.

### Objective 2: Identifying traits that create a liana signal at the leaf, canopy, and stand scales via inverse modeling

**FIGURE 4:**
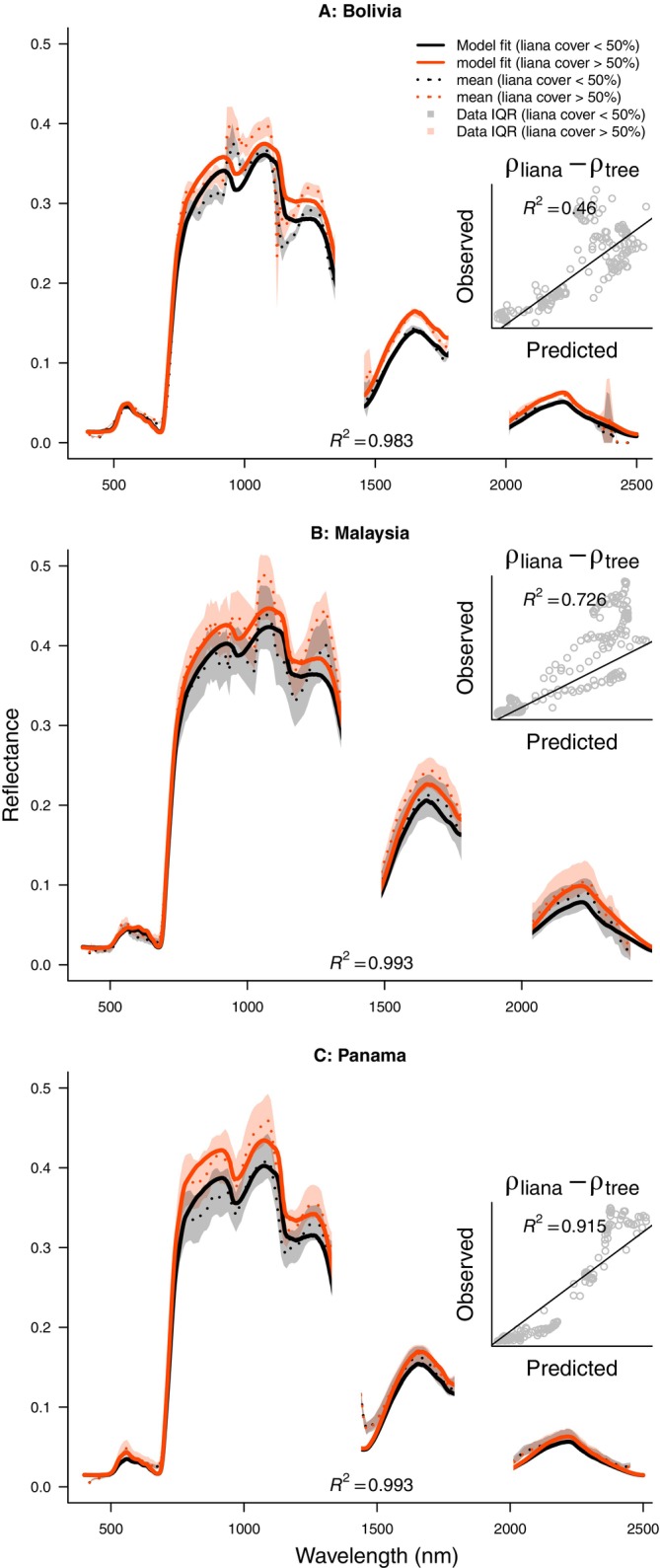
PROSAIL2 model predictions for high and low levels of liana canopy coverage compared with their observed reflectance from airborne and satellite hyperspectral signals. Dotted lines and colored envelopes give mean and inter quartile range (IQR) respectively. Model fits performance was uniformly high, as measured by the coefficient of determination (*R* ^2^ > 0.98). Inset plots show model predictions of the differences in reflectance between high and low liana coverage, which all compared well with the observed difference (*R* ^2^ = 0.46, 0.73, and 0.92), respectively for Bolivia, Malaysia, and Panama). An alternative figure, with reflectance on a log‐scale, is given in Appendix S1: Section S4, Figure S4.

The leaf signal was accurately reproduced by PROSPECTD, exhibiting a low overall error (*R* ^2^ > 0.94 for 5424 individual leaves; Appendix S1: Figure S2). Inverse fits of the PROSAIL2 model to canopy and stand scale data closely approximated the spectral reflectance of heavily infested (>50% cover) and lightly infested canopies (<50%) in Bolivia, Malaysia, and Panama (*R* ^2^ > 0.98; Figure 4A–C). The model also successfully replicated the difference in reflectance between heavily and lightly infested canopies (*R*_lianas_ − *R*_trees_), explaining between 46% and 92% of the variation (inset plots in Figure 4A–C). Model posteriors can be found in Appendix S1: Section S5.

### Objective 3: Model validation

The PROSPECTD model, fitted to leaf‐reflectance data, accurately and unbiasedly replicated independently measured leaf traits such as *C*_ar_, *C*_ab_, *C*_m_, and *C*_w_, outperforming PROSPECT5B (Appendix S1: Section S6), with observed and predicted traits showing agreement ranging from *R* ^2^ = 0.4 to 0.73 (Appendix S1: Figure S8). The leaf structural parameter strongly correlated with leaf thickness (*R* ^2^ = 0.54), suggesting it captured meaningful physical variation.

**FIGURE 5:**
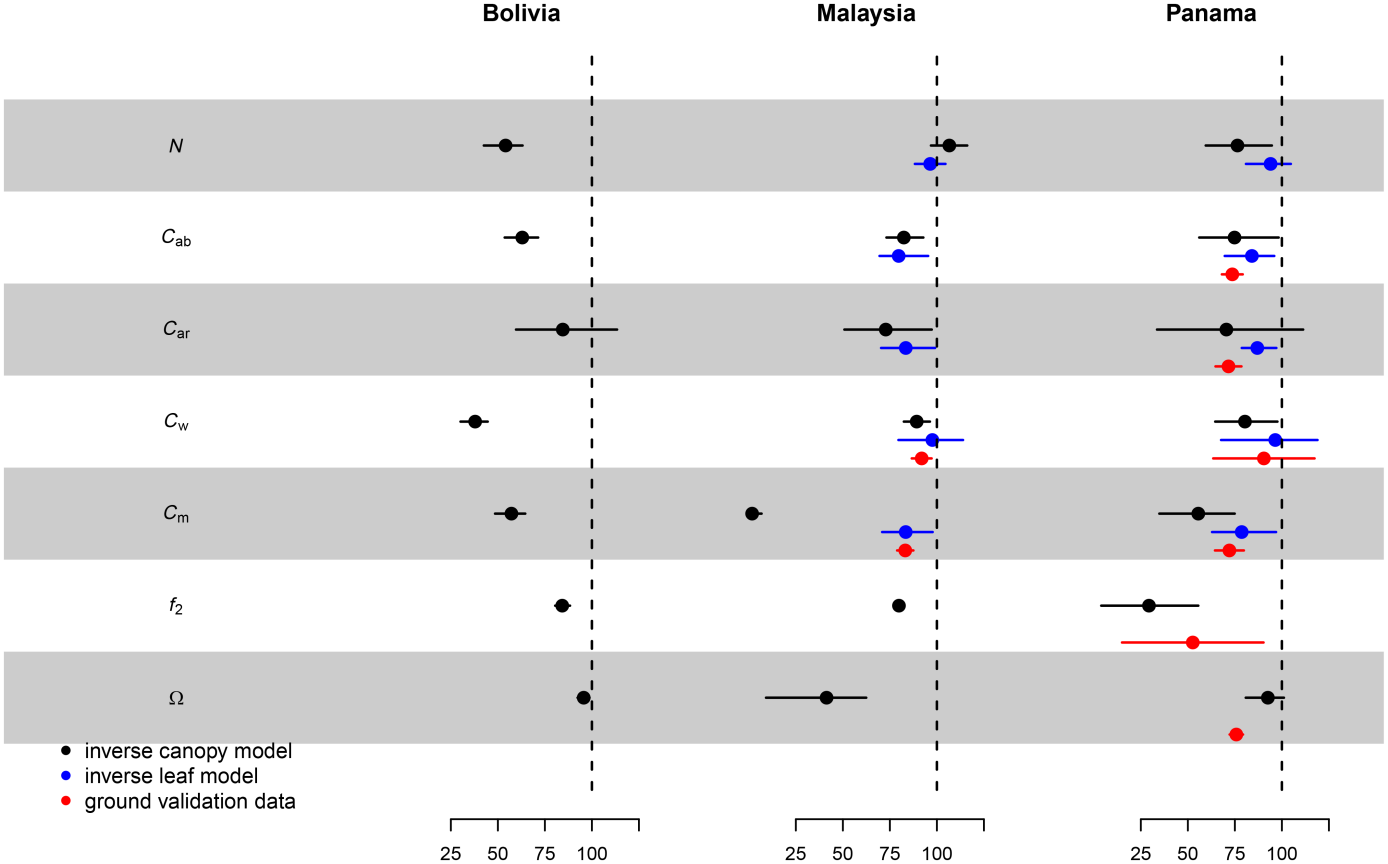
Leaf and canopy traits of liana‐covered and liana‐free forest canopies. Inverse estimates for lianas and trees from full canopy models are compared at each site (black) with independent leaf model data from spectrometers (blue) and, where available, independent field and lab measurements (red). Traits are shown as percentages of posterior mean tree traits for easy comparison, irrespective of units. Absolute values and ground truth comparisons are in Appendix S1: Section S6. The fraction of leaf area index due to lianas is noted as 1 − *f*_2_. Mean values and 95% credible intervals are represented with dots and whiskers. Parameters shared among groups (as soil moisture), or not significantly different between groups for model inversions and ground truth across any sites (*C*_br_) were omitted. Full details on parameters and their posterior distributions are in Appendix S1: Section S5.

Figure 5 depicts the predicted differences between lianas and trees based on inverse fits of PROSAIL2 (black dots and whiskers) for parameters showing nonoverlapping credible intervals (CI) between the liana and tree groupings, at least once across sites. The pattern of differences between lianas and trees in inversely estimated parameters was generally consistent across sites, although not all parameters exhibited significant differences at all sites based on 95% CI (Figure 5). Regarding leaf biophysical and chemical parameters, all models indicated that lianas had more cheaply constructed leaves on average, characterized by thinner leaves, lower photosynthetic pigment content (Chlorophyll and Carotenoids) per unit leaf area, and lower dry mass and water mass per unit leaf area. Note that water mass *C*_w_ (in grams per square meter) should not be equated with leaf water content (in percentage): lianas had higher water content compared with trees (77% vs. 70% on average). Inverse fits with PROSPECTD confirmed the independent predictions of PROSAIL2 whenever leaf‐level spectral data were available (blue dots and whiskers in Figure 5), except for *C*_m_ in Malaysia, which was lower according to PROSAIL2 compared with *C*_m_ estimated from leaf reflectance (PROSPECTD) and direct measurements. However, all estimates of *C*_m_ in Malaysia aligned with the general trend of lower leaf mass per unit area for lianas compared with trees (Figure 5). At the canopy and stand scales, the models predicted that trees contribute an average of 66% (*f*_2_) to the total LAI across sites (Figure 5). Hence, liana leaves contribute 34% (1 − *f*_2_) to the total LAI when a tree crown is fully infested, with site averages ranging from 16% to 55% (Figure 5). Liana leaves were also consistently predicted to have flatter leaf angles, with parameter Ω_1_ being 4%–59% lower than Ω_2_ across sites (Figure 5). On average, leaf angles were 23.5% flatter compared with trees across all three sites.

The inverse fits with the PROSAIL2 model were supported by independently collected verification data where available (red dots in Figure 5). Lab measurements of leaf thickness, chlorophyll, and carotenoids content, dry mass, and water content per unit area for liana leaves were all found to be lower on average for liana leaves (red dots and whiskers in Figure 5). Canopy and stand scale validation data also showed consistency with the model predictions. The estimation of the fraction of PAI attributed to trees versus lianas (*f*_2_ vs. 1 − *f*_2_) using LI‐COR data in plots where lianas were removed showed that trees contribute 65.3% (*f*_2_ = 0.653 ± 0.087 SE) to the PAI when their canopies are infested. Lianas, therefore, contribute on average 34.7% to the total PAI—a value close to the average inverse model estimate (34%). Additionally, the inverse model results predicted flatter leaf angles for all sites (23.5% flatter on average, Figure 5). Measurements conducted in Panama, on 1540 individual leaves from 37 species, confirmed that lianas have 24.8% flatter leaf angles, on average, compared with trees (mean angle of 27.9° ± 0.76 SE vs. 37.1° ± 0.64 SE). Furthermore, field measurements showed that liana leaves have a distinctly flatter distribution of leaf angles compared with trees (Appendix S1: Figure S1, KS test: *D* = 0.26, *p* = 1.9 × 10^−30^).

### Objective 4: What causes the liana signal?

**FIGURE 6:**
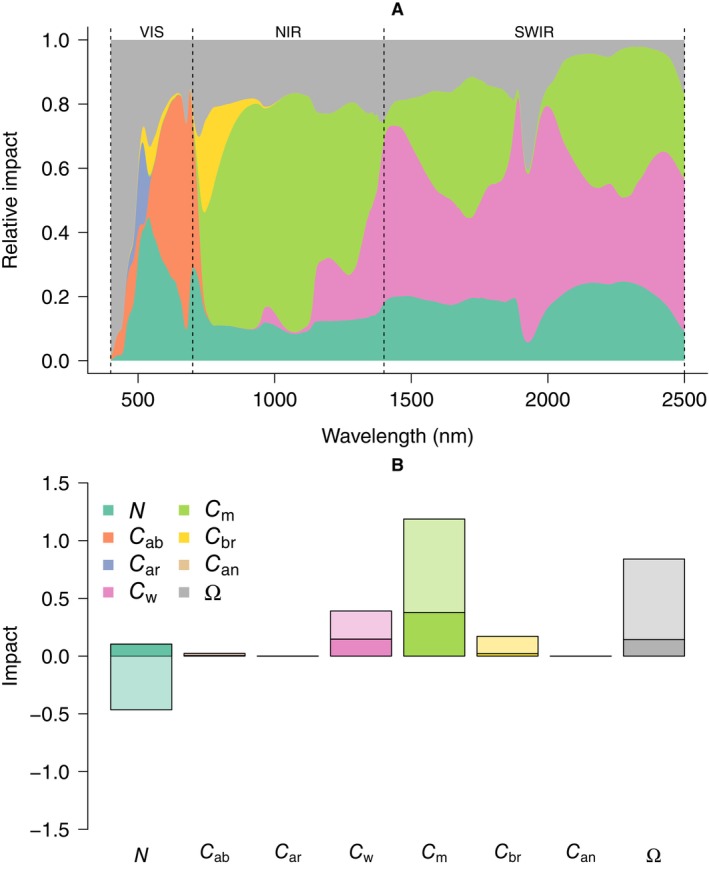
The relative importance of different traits in generating the liana signal across sites. Here, spectral traits of the top layer were initially set as tree traits and gradually replaced with liana traits either one by one (additive) or in pairs (interactive effects). The Kullback–Leiber divergence score was calculated at each iteration. Panel A displays the average relative absolute impact of changing a single trait in the top layer at each wavelength across all sites. Panel B shows the total impact of additive (darker colors) and interactive effects (lighter colors). Positive impact indicates a change toward the liana signal, while negative impact indicates a divergence. Appendix S1: Figure S13 presents the same data for individual regions (Bolivia, Malaysia, and Panama). VIS, visual spectrum; NIR, near infrared; SWIR, short‐wave infrared.

The influence of parameters on the liana spectral distribution varied depending on the specific solar spectrum region (Figure 6A). In the visible spectrum, leaf angles (Ω) and photosynthetic pigment content (*C*_ar_, *C*_ab_) played significant roles, with flatter leaf angles and lower pigment content leading to increased reflectance. In NIR, leaf angles (Ω), leaf mass area (*C*_m_), and leaf structure (*N*) consistently showed importance, although the relative significance of leaf angles (Ω) versus leaf mass area (*C*_m_) differed among sites (Appendix S1: Figure S13A,C,E). Leaf water content (*C*_w_) became increasingly influential with wavelength and dominated the signal in the SWIR (Figure 6A), particularly at the tropical dry site in Bolivia (Appendix S1: Figure S13A). The most influential traits overall were leaf mass area (*C*_m_), leaf angles (Ω), and leaf water content (*C*_w_) (Figure 6B). Leaf angles (Ω) were consistently important across sites, while the importance of *C*_m_ and *C*_w_ varied among sites (Appendix S1: Figure S13B,D,F). Substituting the leaf structural parameter (*N*)—which defines the number of cell‐air interfaces—from trees to lianas resulted in predictions that deviated more from the expected liana spectral distribution (negative contribution), especially when interacting with other parameters (light colors in Figure 6B).

### Objective 5: Theoretical limits to the detection of liana

**FIGURE 7:**
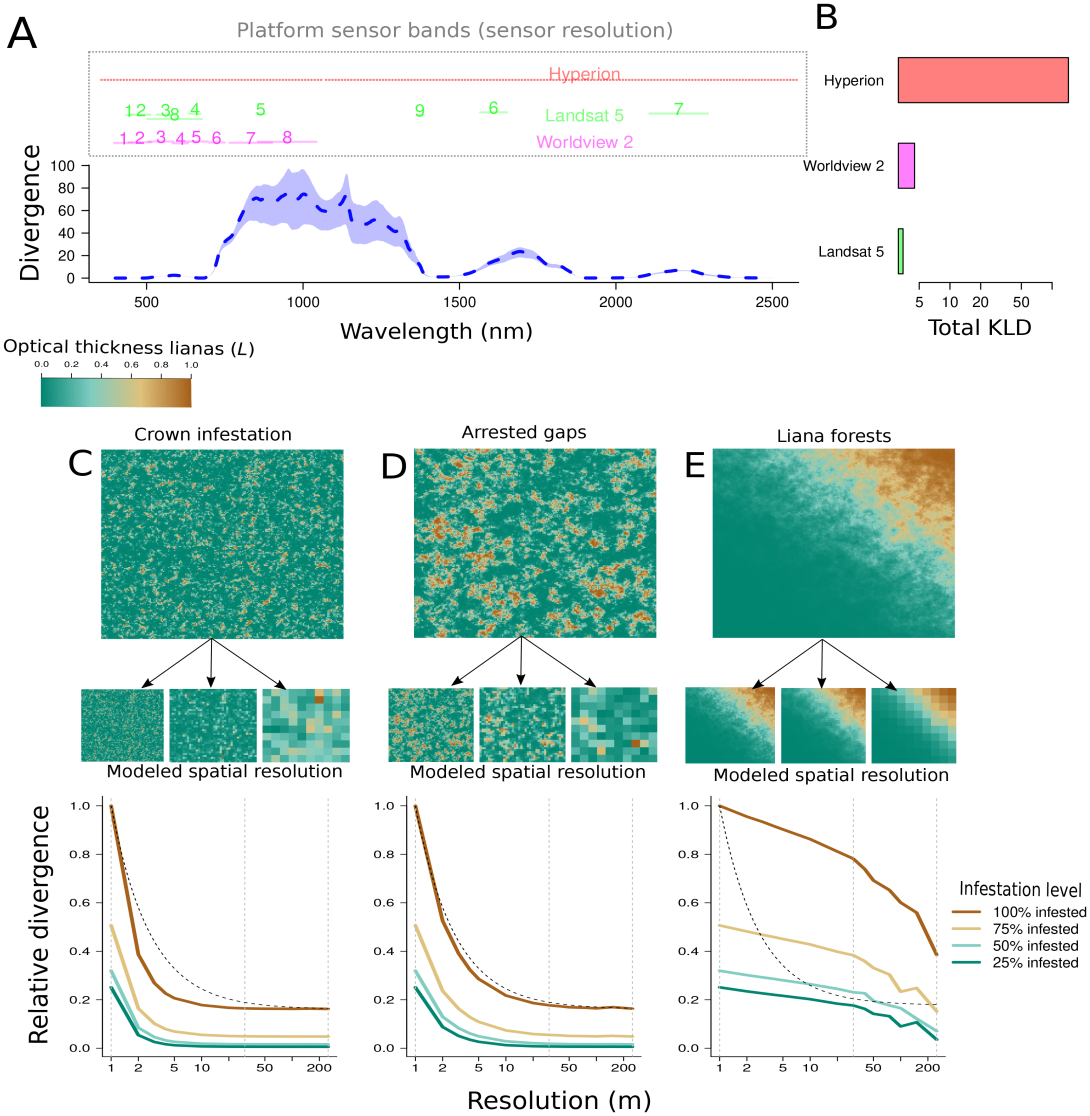
The theoretical capacity of classifiers to distinguish lianas measured with the expected Kullback–Leiber divergence (KLD). Panel A predicts KLD across wavelengths for 100% liana infestation, with model‐posterior based variance estimatimates (colored envelopes), and overlays spectral sampling regions (bands) of various sensors to assess their effectiveness in liana detection. Panel B presents the cumulative KLD for each sensor, with mean divergences of 0.63 (Hyperion), 0.54 (WorldView 2), and 0.45 (Landsat 5). Panels C–E explore the impact of sensor spatial resolution on liana discernibility at different scales: Crown‐scale infestation (C), forest gap arrestation (D), and liana forests (E). They include simulated scenes and resampling results, showing the effect on KLD. Liana cluster sizes in these simulations are 350, 2000, and 30,000 m^2^, corresponding to infestation scales from prior studies (Chandler et al., 2021; Foster et al., 2008; Marvin et al., 2016; Schnitzer et al., 2000; Tymen et al., 2016). The colormap, and line colors in the graphs show different liana infestation levels (L). Gray vertical lines mark different resolutions, and a black dashed line shows the expected KLD decline trend for randomly distributed feature pixels. Each scenario has a roughly equal number of feature pixels.

The KLD between liana‐infested and liana‐free spectral distributions was highest in the NIR and SWIR bands, which are covered by several multispectral spaceborne sensors (Figure 7A). The performance of a sensor in discriminating lianas from trees improved with an increased number of bands within the optimal range (Figure 7B). The spatial resolution of the sensor interacts with liana spatial aggregation (Figure 7C–E). In a crown infestation scenario (liana pixel aggregation ~350 m^2^), the divergence between feature and background decreases rapidly (Figure 7C). For larger spatial aggregation resembling arrested forest succession (2000 m^2^), the divergence dropped at a similar pace to random feature pixels (Figure 7D). For highly aggregated lianas across extensive forest areas (~30,000 m^2^), the divergence decreased slower than the random expectation.

## DISCUSSION

We demonstrate a unique reflectance distribution in liana‐infested canopies and forest stands, showing higher reflectance than liana‐free areas (Figures 1 and 4), particularly in the NIR and SWIR spectrum sections (Figure 7). This distinct canopy‐scale reflectance distribution, observed across four globally dispersed tropical forest sites (Figure 1), has implications for our understanding of liana evolution and forest energy budgets and provides a basis for expanded research on lianas at pantropical scales. However, the traits underpinning this distribution might not be exclusive to lianas, posing a clear risk of confusion with other phenomena.

### The mechanisms behind the liana spectral signal

Our analysis reveals a geographically consistent liana reflectance distribution compared with trees across sites, observable at larger scales (≥1 m^2^; Figures 1 and 4), but absent in leaf‐scale reflectance (<<1m^2^; Figure 3). Model predictions indicate leaf traits alone do not account for the consistent reflectance difference between liana and tree leaves across sites (Figure 2A and Appendix S1: Figure S12). Previous research confirms varying leaf reflectance distributions and optical traits for liana compared with tree species and sites (Asner & Martin, 2012; Medina‐Vega, Bongers, Poorter, et al., 2021; Sánchez‐Azofeifa, Castro, et al., 2009). Therefore, we conclude that leaf reflection alone is unlikely to be critical in determining liana canopy reflectance. In contrast, differences in leaf traits between lianas and trees do affect transmission and absorption profiles (Figure 2) and, by interacting with canopy traits, likely drive the distinct reflectance distribution observed across multiple sites and platforms (Figures 1 and 4). Our findings are supported by RTM inversions at the canopy and stand scales (Figures 5 and 6).

Differences in leaf traits between lianas and trees yield consistent shifts in leaf absorption and transmission distributions (Figure 2), and we predict lower leaf absorption and increased transmission, on average, for liana leaves at the canopy scale across evaluated sites (Appendix S1: Figure S12). Plausible mechanisms for the distinct canopy‐scale liana reflectance distribution involve reduced light extinction rates in liana‐infested canopies. In addition, our model experiment highlights the importance of flatter leaf angles in liana leaves compared with host trees in generating the canopy‐scale liana reflectance distribution (Figures 5 and 6). We propose a two‐component mechanism for the “liana signal” (Figure 7A): (1) higher projected leaf area, which, at equal leaf reflectance, leads to increased reflected energy at the canopy level, and (2) multiple scattering from greater transmission and lower leaf absorption, which indirectly increases canopy reflectance. The latter is an effect similar to what is hypothesized to cause “canopy greening” (Wu et al., 2018); seasonal increases in spectral vegetation indices observed above aseasonal rainforests.

Building on our mechanistic models, we can derive theoretical expectations for the two‐component mechanisms that generate the liana canopy‐level reflectance distribution. The first component arises as lianas retain, on average ~24% flatter leaf angles (27.9° vs. 37.8°), which should result in roughly an 11% increase in projected leaf area as seen from a zenith angle and a proportional increase in the reflected energy. The second “multiple scattering” component arises from liana leaves having lower average absorption and higher transmission (Figure 3 & Appendix S1: Figure S12). This results in slower decay of energy (Appendix S1: Section S9) and increased scattering, including in the observer's direction, leading to higher reflectance when integrated across the canopy. Multiple scattering also explains the regions where the liana canopy‐reflectance distribution differs most from trees. Since the projected leaf area does not change with wavelength, its importance is relatively uniform across the spectrum (Figure 6A, parameter Ω). The importance of multiple scattering, however, peaks when leaf transmission is maximized, in the SWIR and NIR parts of the spectrum. This corresponds to the point of maximal discernibility of the liana reflectance distribution (Figure 7), and where leaf traits appear the most influential in our sensitivity analysis (Figure 6).

In conclusion, a distinct liana canopy‐reflectance distribution emerges from interactions between multiple leaves within canopies. This multi‐leaf dependency explains why the signal is observed at the canopy scale (Figures 1 and 4; Chandler et al., 2021; Foster et al., 2008; Tymen et al., 2016; Waite et al., 2019) and the lack of a consistent signal when only leaf reflectance is considered (Figure 3, Sánchez‐Azofeifa, Castro, et al., 2009).

### Ecological consequences of the liana spectral signal

By increasing forest canopy albedo (Figure 2), lianas have the potential to impact forest energy budgets and understory light conditions. As tropical forests are typically light‐limited and liana abundance is increasing across the globe (Schnitzer & Bongers, 2011; Wright et al., 2015; Rueda‐Trujillo et al., 2024), liana proliferation could have broader impacts beyond their typically mentioned impacts on carbon budgets. For instance, Meunier et al. (2021) integrated liana optical properties into the ED2 dynamic vegetation model suggesting several consequences. With approximately 25% liana leaf area, the model predicted an increase in reflected energy in the PAR (17.1%) and IR (11.7%) spectrum. This resulted in a reduction of 3.6 Wm^−2^ in absorbed energy, leading to darker understories (up to 50% darker) and slightly cooler soils (−0.5°C in the topsoil). These findings highlight the broader impacts of the liana spectral signal on forest functioning, including temperature‐dependent processes such as metabolic respiration and photosynthesis. Our results support Meunier et al.'s findings and indicate a global characteristic of higher albedo in liana‐infested areas. We believe this shows a direct and urgent need for empirical validation of these model predictions to better understand the potential long‐term changes in forested ecosystems with increasing liana prevalence.

### Robust detection of lianas

Our simulations highlight two primary insights regarding liana detection. First, many sensor platforms often miss optimal bands distinguishing lianas from trees. Satellite platforms might possess just one optimal band (e.g., Landsat, Figure 7A). Second, as crown infestation levels increase, the liana spectral distribution decays non‐linearly (Figure 7C–E). High spatial resolution is essential for detecting lianas within tree crowns, where signal weakening of small clumps, due to spectral mixing, occurs faster than expected if infested pixels were randomly distributed (Figure 7C). This means that moderate‐scale platforms (around 20–30 m resolution) are less suitable for detecting crown infestations but should be able to identify larger liana‐infested regions where the signal decays slower as sensor spatial resolution decreases (Figure 7E). These two simulation findings echo patterns from earlier studies. Moderate resolution platforms like Hyperion and Landsat effectively identified large liana‐dominated areas (Foster et al., 2008; Tymen et al., 2016), whereas high‐resolution hyperspectral images were best for detecting heavy tree crown infestations (Chandler et al., 2021; Marvin et al., 2016). Hence, our findings can inform future liana remote sensing research and sensor selection. However, caution is advised as this analysis, being a “best‐case scenario” overlooks atmospheric factors potentially weakening satellite signals (Landgrebe & Makaret, 1986).

#### Confounding variables

Our analysis of the liana spectral distribution prompts careful use of classifiers due to potential signal confusion. Notable confounders are:

1. Leaf phenology. Seasonal shifts in phenology can confound classification. Like lianas, younger tree leaves exhibit lower *C*_m_, *C*_w_, and pigment concentrations, causing them to resemble liana signals by being more transmissive (Wu et al., 2018).
2. Tree life history. Light‐demanding species possess flatter leaf angles and thinner leaves, risking liana misclassification. Concurrently, liana infestation can obscure tree signals, causing mapping inaccuracies in studies aiming to differentiate tree species (Zhang et al., 2006).
3. Topography and sun‐sensor geometry. Areas sloping toward the sun may appear brighter across the spectrum, akin to liana reflectance, potentially causing an automated classifier to misidentify two pixels only varying in aspect as having different levels of infestation.
4. Climate. Some key leaf‐trait differences between lianas and trees identified here, lower *C*_m_ and *C*_w_, remain but become less pronounced in wetter climates (Asner & Martin, 2012; Medina‐Vega, Bongers, Schnitzer, & Sterck, 2021; Sánchez‐Azofeifa, Castro, et al., 2009). As these traits are mostly responsible for the maximal divergent signal from trees in the NIR and SWIR portions (Figure 7), the spectral difference between liana‐infested and free canopies may also become less pronounced.

These confounding factors in detecting lianas can be addressed uniquely. Seasonal leaf phenology changes contrast with lianas' longer residency time in canopies, aiding detection, especially in dry forests where lianas retain leaves longer than their deciduous host trees (Schnitzer, 2005). Light‐demanding tree species, less prone to liana infestation and with fewer leaf layers (Kitajima, 1994; Visser, De Kroon, et al., 2018), can be distinguished using lidar structural data alongside spectral information (sensu Rao et al., 2020). This approach is particularly relevant given the substantial structural changes caused by liana infestation, such as canopy wood area index alteration (Sánchez‐Azofeifa, Kalácska, et al., 2009). Furthermore, topography and sun‐sensor geometry issues might be mitigated with multiangle sensors (e.g. CHRIS Proba‐1) or matching‐resolution digital elevation models. Thus, automated classifiers should be applied with caution over large areas, ensuring robustness against these and other confounding factors.

### Caveats and limitations

The main result from model inversion at three sites was backed up by independent measurements in the field and previous studies (Mello et al., 2020; Sánchez‐ Azofeifa & Castro‐Esau, 2007). Separate measurements of leaf reflectance and in situ measurements of leaf traits and angles confirmed that lianas tend to have relatively flatter leaf angles and more cheaply constructed leaves with less dry matter and pigments. However, the model's predictions were not always perfectly in line with ground truth data. On an absolute scale, ground measurements of mean leaf angles were lower than those predicted by PROSAIL2 (Appendix S1: Section S6). One explanation for this is that we do not consider the effect of leaf clustering on stems and branches in our field campaign, while the PROSAIL2 result is more likely to reflect the integrated angles of leaves clustered on stems and branches. Direct measurements (i.e., not derived from remotely sensed data) on leaf angles, stems, and branches collected from multiple sites would confirm the robustness of this result.

## CONCLUSIONS

Our work reveals a unique spectral reflectance signature for lianas at the canopy scale, detectable by aerial and spaceborne sensors. Models attribute the signature to lianas' larger projected leaf area, lower leaf light absorption and higher transmission, leading to increased canopy‐level light scattering. We observe the signal, characterized by higher forest albedo, in four distinct tropical sites, suggesting a consistent global pattern. If further confirmed, this could significantly impact our understanding of liana proliferation's effects on forest energy budgets and understory dynamics. The identification of a general spectral signal for liana infestation across sites underscores the opportunity for developing classifiers for quantifying liana abundance across large spatial scales. Our successful use of RTMs also highlights the risk for confounding and the need for creating and validating robust, potentially physics‐informed classifiers. In conclusion, fertile ground exists for future research into the liana spectral signal, its robust detection from remote sensing platforms, and the ecological consequences of liana proliferation.

## CONFLICT OF INTEREST STATEMENT

The authors declare no conflicts of interest.

## Supporting information


Appendix S1.

## Data Availability

All 17 datasets are publicly accessible (links in Appendix S1: Section S1.1). The open‐source model code is available as official R package ccrtm (Visser, 2020).
