# Supplementary material for: When can we detect lianas from space? Toward a mechanistic understanding of liana‐infested forest optics
Source: Ecology. 2025 Apr 27;106(4):e70082. doi: 10.1002/ecy.70082 (PMC12035525; doi:10.1002/ecy.70082)
Supplement: Supplementary file 1 — Appendix S1. [file ECY-106-e70082-s001.pdf]

Appendix S1: When can we detect lianas from space? Towards a mechanistic understanding of liana-infested forest optics

Marco D. Visser, Matteo Detto, Félicien Meunier, Jin Wu, Jane R. Foster, David C. Marvin, Peter M. van Bodegom, Boris Bongalov, Matheus Henrique Nunes, David Coomes, Hans Verbeeck, J. Antonio Guzmán Q, Arturo Sanchez-Azofeifa, Chris J. Chandler, Geertje M.F van der Heijden, Doreen S. Boyd, Giles M. Foody, Mark E.J. Cutler, Eben N. Broadbent, Shawn P. Serbin, Stefan Schnitzer, M. Elizabeth Rodríguez-Ronderos, Frank Sterck, José A. Medina-Vega & Stephen W. Pacala

March 24, 2025

Journal: *Ecology*

Contents

Datasets 3
Data sources . . . 5
Data descriptions . . . 6

Bayesian model fitting 15

Model robustness analyses 19

Leaf reflectance data and leaf model fits 20
Leaf reflectance data . . . 20
Summary of leaf model fit statistics . . . 22
Canopy model fits . . . 24

Posterior distributions 25

Absolute Validation 29

Leaf profiles 33

Model experiment per site 35

Multiple scattering 37

References 39

Section S1 Datasets

Dataset numbering corresponds to table S1 and in the main text. References are supplied with a doi if they are not referenced in the main text. In addition, a full reference list for the supporting information is given in section S10 (references).

Spectral datasets, location and sensor information

	Scale	spatial extent*	Locations **	Year	Species (vines)	Samples	platform	Sensor
1	Leaf	$< 10\ cm^2$	Ispra, Italy	1993	46 (3)	330 leaves	Spectroradiometer	PerkinElmer LAMBDA-19
2		$< 10\ cm^2$	Angers, France	2004	43 (5)	276 leaves	Spectroradiometer	ASD FieldSpec-FR
3		$< 10\ cm^2$	SLZ & PNM, Panama	2004	40 (27)	864 leaves	Spectroradiometer	Unispect PPSystems
4		$< 10\ cm^2$	SLZ & PNM, Panama	2016	6	248 leaves	Spectroradiometer	SVC HR1024i
5		$< 10\ cm^2$	SLZ & PNM, Panama	2017	28	852 leaves	Spectroradiometer	SVC HR1024i
6		$< 10\ cm^2$	CAM, CAR, FLMM, SB, UPR, Puerto Rico	2017	11	104 leaves	Spectroradiometer	SVC HR1024i
7		$< 10\ cm^2$	TNF, Brazil	2012	16	166 leaves	Spectroradiometer	ASD FieldSpec Pro
8		$< 10\ cm^2$	TNF, Brazil	2017	11	759 leaves	Spectroradiometer	ASD FieldSpec Pro
9		$< 10\ cm^2$	VAR, C. & S. America	2017	214(20)	650 leaves	Spectroradiometer	ASD FieldSpec 3 & SVC HR1024i
10		$< 10\ cm^2$	DVCA, Malaysia	2015	282	683 branches	Spectroradiometer	ASD FieldSpec 4
11		$< 10\ cm^2$	KSFR, Malaysia	2016	67 (20)	492 leaves	Spectroradiometer	ASD FieldSpec Pro
12	Canopy	$1\ m^2$	DVCA, Malaysia	2014	NA	455 crowns	Airborne	Asia-Fenix
13		$2\ m^2$	BCNM, Panama	2012	NA	544 crowns	Airborne	AToMS
14	Stand	$30\ m^2$	NER,French Guina	2006	NA	450 pixels	Satellite	Landsat
15		$30\ m^2$	NKM, Bolivia	2001	NA	8164 pixels	Satellite	Hyperion

Table S1: *Spectral sampling area is typically bewteen .6 to 3.5 cm^2 depending on leaf clip or artificial light specifications. **Abreviations alphabetically: Barro Colorado Nature Monument (BCNM), Cambalache State Forest (CAM), Carite State Forest (CAR), Danum Valley Conservation Area (DVCA), Fundacion Luis Munoz Marin (FLMM), International Institute of Tropical Forestry (UPR), Kabili-Sepilok Forest Reserve (KSFR), Nouragues Ecological Research Station (NER), Noel Kempff Mercado National Park (NKM), Parque Nacional San Lorenzo (SLZ), Parque Natural Metropolitan (PNM), Sabana (SB), Tapajos National Forest (TNF), and various sites throughout Central and South America (VAR).

Section S1.1 Data sources

1. (LOPEX), ECOSIS library: https://ecosis.org/package/leaf-optical-properties-experiment-database--lopex93-
2. (ANGERS), ECOSIS library: https://ecosis.org/package/angers-leaf-optical-properties-database--2003-
3. (Sánchez-Azofeifa *et al.* 2009, Sánchez-Azofeifa 2025), Harvard Dataverse: https://doi.org/10.7910/DVN/OG95TV
4. (NGEE-TROPICS), LBL library: http://dx.doi.org/10.15486/ngt/1475180
5. (NGEE-TROPICS), LBL library: http://dx.doi.org/10.15486/ngt/1478523
6. (NGEE-TROPICS), LBL library: http://dx.doi.org/10.15486/ngt/1475180
7. (Wu et al. 2012,2017), ECOSIS library: https://ecosis.org/package/2012-leaf-reflectance-spectra-of-tropical-trees-in-tapajos-national-forest
8. (Wu et al. 2018, Visser et al. 2025), available from Dryad: https://doi.org/10.5061/dryad.xpnvx0krs.
9. (Meireles et al. 2020): http://dx.doi.org/10.6084/m9.figshare.12449147.v1
10. (Nunes et al 2019, doi: 10.1088/1748-9326/ab2eae): available from Dryad: https://doi.org/10.5061/dryad.xpnvx0krs
11. (Sepilok Forest Reserve spectral data), available from Dryad: https://doi.org/10.5061/dryad.xpnvx0krs.
12. (Chandler et al 2020, NERC Airborne Research and Survey Facility 2019), library: http://dx.doi.org/10.5285/c708cad9950c45b1af0d4e9ca944f09a
13. (Marvin et al 2016, Visser, et al. 2025), available from Dryad: https://doi.org/10.5061/dryad.xpnvx0krs
14. (Tymen et al 2016,Tymen et al 2019): available from Dryad: https://doi.org/10.5061/dryad.1pc19
15. (Foster et al 2008; U.S. Geological Survey 2019), EO-1 Hyperion data: https://doi.org/10.5066/P9JXHMO2,image with ID EO1H2300702001248112PP_SGS_01 from 2001-09-05 is downloadable via https://earthexplorer.usgs.gov.
16. (Rodríguez-Ronderos, et al 2016, Visser, et al. 2025), available from Dryad: https://doi.org/10.5061/dryad.xpnvx0krs.

17. (Visser and Detto, Visser, et al. 2025), leaf angle data, available from Dryad: `https://doi.org/10.5061/dryad.xpnvx0krs`.

Section S1.2 Data descriptions

- **Dataset 1 - 2**: Both datasets 1 and 2 are well known in the remote sensing community as the LOPEX and ANGERS datasets respectively. Leaf biochemical and spectral measurements were conducted for both datasets.

  For LOPEX, a total of 330 leaves from 46 species were collected in 1993 at the Joint Research Centre at Ispra in Italy. The optical measurements were performed on the leaf blades using a Perkin Elmer Lambda 19 double-beam spectrophotometer. The ANGERS dataset comprised 276 leaves from 43 species collected in June 2003 at the INRA Agricultural Experiment Station of Angers in France. In this case, the optical measurements were conducted using a FieldSpec-FR portable Spectroradiometer. In both datasets, an integrating sphere coated with BaSO4 was used, enabling the measurement of reflectance and transmittance spectra within the range of 350 to 2500 nm.

  Following the spectral measurements, leaf discs with a diameter of 5 mm were sampled to determine biochemical content. Pigments were extracted by grinding the leaf discs in a chilled mortar and using 95% ethanol (v/v). Water content and dry matter content ($g.cm^{-2}$) were calculated by weighing and averaging three discs with a diameter of 10 mm from fresh and dry samples. The fresh weight was measured before placing the leaf samples in a drying oven at 85 $\circ$ C for 48 hours. After drying, the samples were reweighed to determine the dry weight and water content.

  Additional details on the LOPEX dataset can be found in (Hosgood et al., 1994)[1], and for the ANGERS dataset, refer to Pavan et al. (2004)[2].

- **Dataset 3**: During the rainy season in August 2004, sun leaves from ten individuals of the most abundant species within the crane arm's reach were collected. The sampling followed established protocols, resulting in a total of 26 liana and 9 tree species sampled from PNM, and 9 liana and 9 tree species from FS. Each species had a maximum of 10 leaves collected, in accordance with Panamanian National Environment Authority guidelines. The leaves, free of epiphytes, mosses, and galls, were immediately stored in sealable plastic bags with moist paper towels and then placed in a larger ice-containing black plastic bag.

  Spectral reflectance measurements (400 to 1100 nm) were taken on the day of collection using a portable spectrometer. This method captures

[1] Hosgood, B., Jacquemoud, S., Andreoli, G., Verdebout, J., Pedrini, G., Schmuck, G., 1994. Leaf Optical Properties EXperiment 93 (LOPEX93). Eur. Comm. Jt. Res. Centre, Inst. Remote Sens. Appl. Rep. EUR 16095 EN 11.

[2] Pavan, G., Jacquemoud, S., De Rosny, G., Rambaut, J.-P., Frangi, J.-P., Bidel, L.P.R., 2004. RAMIS: A NEW PORTABLE FIELD RADIOMETER TO ESTIMATE LEAF BIOCHEMICAL CONTENT.

leaf structure, pigment sizes, and water content but not rapid photochemical changes. A bifurcated fiber optic and leaf clip were used, and the data included visible/near-infrared reflectance. Leaf cores were then preserved for pigment analysis. Additionally, using a barium-sulfate-coated integrating sphere, diffuse transmittance and reflectance were measured for five leaves per species, with absorptance estimated as the complement of these values. These data were aggregated for further analysis at the structural group level.

Leaf thickness was measured on five mature leaves from different individuals of each species. The average thickness was calculated from six points near the apex, middle, and base using a leaf thickness micrometer. These measurements were made within two hours of collection. Additionally, the fresh and dry weights of the leaves were determined, with dry weight measured after drying at 60 ˚C for 36 hours. Nitrogen and phosphorus concentrations were estimated using a combustion elemental analyzer and colorimetric analysis, respectively. Specific leaf area (SLA) was calculated as the ratio of fresh surface area to dry weight.

Detials can be found in Sánchez-Azofeifa *et al.* 2009.

- **Dataset 4, 5 & 6**: A total of 27 canopy tree species from two sites in Panama were selected for intensive field measurements of leaf reflectance spectra and traits in 2016 and 2017.

  The sampled leaves were categorized into two main age classes: immature leaves (less than 2 months old), which encompassed leaves from emergence to fully expanded but not fully green, thickened, or physiologically matured, and mature leaves (2 months or older). This age classification aligns with similar categories proposed by Coley (1983, doi:10.2307/1942495) and Wu et al. (2016,doi: 10.1111/nph.14051). However, unlike Wu et al. (2016), where three age categories (young, mature, and old) were used, our study combined the mature and old age classes into a single mature age class.

  Field measurements were conducted during the dry seasons of 2016 and 2017 on sunlit upper canopy foliage in Panama. In the 2016 field campaigns, which took place in mid-February and mid-April, we sampled the dominant leaf class(es) from eight trees at each site. In February 2017, the measurements included both age classes if they were present within the top meter of a sunlit branch from four canopy tree species at the SLZ site.

  Leaf reflectance at the Panamanian sites was measured using a Spectra Vista Corporation (SVC) HR-1024i spectroradiometer (SVC, Poughkeepsie, NY, USA). The spectral range of the instrument was 350-2500 nm, with a spectral resolution of 3.5 nm at 700 nm, 9.5 nm at 1500 nm, and 6.5 nm at 2100 nm. The measurements were conducted with the SVC LC-RP-Pro foreoptic, and all reflectance measurements were referenced

against a 99% Spectralon reflectance standard. Details given in Serbin *et al.* (2019, doi: 10.1111/nph.16123)

- **Dataset 7 & 8**: Measurements of leaf full-spectrum reflectance and transmittance were conducted across 54 tropical tree species, covering the wavelength range of 350-2500 nm. The dataset includes leaves collected from both fully sunlit and shaded canopy strata, as well as leaves representing different age categories, including young, mature, old, and senescent leaves. For each sample, information on the relative age estimate, leaf canopy position, and sample number is provided.

  The data was collected as part of the 2017 NGEE-Tropics / NASA G-LiHT airborne campaign. Additional datasets related to this study contain details such as sample photographs, leaf traits (e.g., leaf mass per area (LMA), water content), and leaf carbon and nitrogen measurements.

  Leaf reflectance was measured using a Spectral Evolution PSR+ full-range spectroradiometer. To perform the measurements, a custom fiber optic connected to a Spectra Vista Corporation LC-RP-Pro foreoptic was utilized. All reflectance measurements were referenced against a 99.9% Spectralon reflectance standard. More detauls given in Wu *et al.* (2016,doi: 10.1111/nph.14051) and Wu *et al.* (2017).

- **Dataset 9**: Meireles et al. (2020) compiled a comprehensive dataset comprising over 16,000 leaf-level reflectance spectra ranging from 400 to 2400 nm. This dataset encompassed 544 seed plant species found in temperate and tropical regions across the Americas and Europe (Fig. 1 in maintext). The included spectra were solely derived from mature, sun-exposed leaves measured during the spring or summer seasons. In our study, we utilized the aforementioned dataset, focusing on a subset of 650 leaves from 214 tropical trees and lianas identified in the dataset using tables provided by Martin and Asner (2008).

  The data contains leaf spectra, obtained from two full-range field spectroradiometers: an ASD FieldSpec 3 (Analytical Spectral Devices, Boulder, CO, USA) and an SVC HR-1024i (Spectra Vista Corp., Poughkeepsie, NY, USA) with measurements were conducted using leaf clips and artificial light sources. Processing details are given in Meireles et al. (2020), and include harmonization among sensors.

- **Dataset 10**: In all field surveys a random selection of five leaves attached to the branches was made, excluding damaged and young leaves. Reflectance spectra spanning the range of 350-2500 nm were acquired using a FieldSpec 4 spectroradiometer, manufactured by Analytical Spectral Devices (ASD) based in Boulder, Colorado, USA.

  To ensure accurate measurements, the spectroradiometer's contact probe was securely mounted on a clamp and pressed firmly against the sample, while a black background was employed to eliminate any external light

interference. The spectral measurements were taken at the midpoint between the main vein and the leaf edge, approximately halfway between the petiole and the leaf tip. The abaxial surface of the leaf faced towards the probe during measurement.

To maintain calibration, readings were periodically adjusted against a Spectralon white reference panel after every five samples. In all subsequent statistical analyses, the spectral data were trimmed to the 400-2500 nm range, and the mean reflectance values from the five spectra per branch were utilized. More details in Nunes *et al.*(2019, doi: `10.1088/1748-9326/ab2eae`)

- **Dataset 11**: Nine 4-ha plots were established in Sepilok between 2000 and 2001. The most recent survey in 2014 included the measurement and identification of all trees with a diameter at breast height (DBH) of 5 cm or more, with 91% identified to the species level. In a 0.25-hectare subplot within each plot, liana abundance, biomass, and diversity were measured for stems over 0.5 cm DBH. Sampling of tree and liana branches occurred in April-May 2016, resulting in 54 tree and 23 liana species sampled from 179 individuals. Common tree species accounted for over 50% of the total basal area, while the sampled liana species represented over 50% of the liana basal area.

  To sample sunlit branches, researchers collected small branches from accessible trees and lianas in the forest canopy, including neighboring crowns of less abundant species for larger trees. The branches were immediately cut underwater, preserved in a water-filled plastic enclosure, and stored in a closed Styrofoam box with ice packs. Bulk leaf samples were collected and dried for elemental analysis. Measurements of traits were conducted in close proximity to the forest edge at the Phytochemistry Laboratory of the Sabah Forest Research Centre.

  For each collected branch, three clean leaves were selected for hyperspectral reflectance measurements. The branch was hydrated overnight, and the weight of three leaves was measured the next morning. The leaves were scanned for area determination and then dried in an electric oven. Elemental concentrations and stable isotope ratios were analyzed using a Costtech Elemental Analyser attached to a Thermo DELTA V mass spectroradiometer.

  Hyperspectral reflectance signatures were measured using a FieldSpec Pro spectroradiometer equipped with a contact reflectance probe and an integrated light source. The spectroradiometer employed three sensors with shared optics, covering different wavelength ranges. To ensure accurate measurements, leaf samples were placed on a low reflectance black background, and a specific procedure was followed to eliminate stray light and account for drift in the illumination source and sensor using dark current and white reference measurements (Chavana, 2017, doi: 10.1111/nph.13853).

Dataset 12: In November 2014, the UK Natural Environmental Research Council's Airborne Research Facility (NERC-ARF) collected manned airborne hyperspectral data over a primary forest area of approximately 2083 ha. The data were acquired using a Dornier 228-201 aircraft, flying at speeds ranging from 127 to 139 knots at altitudes of 2335-2429 m. A total of 10 flightlines were flown, capturing detailed information about the forest (Chandler, 2020).

Hyperspectral imagery was collected using a FENIX sensor with a spatial resolution of 3 m. The data were calibrated in collaboration with the NERC Field Spectroscopy Facility. Radiometric corrections were applied to the dataset, removing bands with missing or overly saturated data. ENVI FLAASH Atmospheric Correction was used for atmospheric correction. To address variations in reflectance values between flightlines, spectral values were adjusted based on the difference in reflectance between overlapping pixels from adjacent flightlines. A Standardized Principal Component Analysis (SPCA) was performed to reduce the dimensionality of the data, retaining eight principal components that explained over 99% of the variation.

Two datasets were collected for the liana canopy cover survey: ground-based below-the-canopy data and UAV-based above-the-canopy data. Ground data were collected between 2017 and 2019, involving the visual delineation of individual tree crowns using LiDAR data uploaded to a tablet computer connected to a GPS receiver. The percentage of liana infestation within each tree crown was estimated independently by field teams through mutual agreement. UAV-derived imagery was used to collect liana infestation data at the tree-level and hyperspectral pixel-scale. A DJI Phantom 4 Advanced quadcopter with an integrated RGB camera was employed for data acquisition, with geo-tagged images processed to generate a single orthorectified image. Individual tree crowns were identified, and liana infestation was estimated by visually assessing the percentage of a pixel covered by lianas within crown boundaries. For additional information, please refer to Chandler (2020).

Dataset 13:Airborne collection of imagery over the Gigante Peninsula in Barro Colorado National Monument in the Republic of Panama occurred in the dry season of February 2012. The Carnegie Airborne Observatory Airborne Taxonomic Mapping System (AToMS) acquired high-resolution data of the site with an integrated full-spectral range (visible-to-shortwave infrared) imaging spectroradiometer (Asner et al., 2012, doi: 10.1016/j.rse.2012.06.012). The AToMS visible-to-shortwave infrared imaging spectroradiometer (VSWIR) measures spectral radiance in 481 contiguous channels spanning the 252-2648 nm wavelength range. At a flight altitude of 2000 m, the VSWIR data collection provided a ground sampling distance of 2.0 m throughout the study landscape. VSWIR spectroradiometer data were radiometrically corrected from raw digital number (DN) values to radiance (W sr^{-1} m^{-2} nm^{-1}) using a flat-field correction,

radiometric calibration coefficients, and spectral calibration data collected in the laboratory, with further post-processing described in detail by Asner et al. (2012). Reflectance imagery was corrected for cross-track brightness gradients using a bidirectional reflectance distribution function. Individual tree crown field assessments were collected in July and August of 2013. Further details on imagery and field data acquisition and processing can be found in Marvin et al. (2016).

- **Dataset 14**: Tymen et al (2016) supply the georeferenced locations of forests heavily infested by lianas (hereafter "liana forest") which was delineated using lidar, aerial photography and a ground census. We obtained a georeferenced 30-m resolution Landsat Thematic Mapper image (L1TP) acquired on October 8 2006 of the study area. The image was atmospherically corrected using the FLAASH algorithm in ENVI 5.5.

- **Dataset 15**: The obtained Hyperion image underwent several checks and analyses. Firstly, a minimum noise fraction transform (MNF) was performed to detect smile, resulting in 25 MNF bands based on eigenvalues greater than or equal to 3 (Goodenough et al., 2003, doi: `10.1109/TGRS.2003.813214`). Smile was observed in the first two MNF bands, noticeable through the brightness gradient (Goodenough et al., 2003). To mitigate the inherent "striping" in the Hyperion data and address the smile effect, a "destreaking" algorithm was applied. This algorithm normalized the pixel values in each sample to the overall mean and standard deviation of all samples (Datt et al., 2003, doi: `10.1109/TGRS.2003.813206`). Before destreaking, specific areas such as clouds, cloud shadows, and background pixel values resulting from VNIR and SWIR coalignment were masked in the 196-band image. Masks were generated using pixel value thresholds in three bands to exclude values that could bias the destreaking results. The MNF transform was then applied to the destreaked image, resulting in a reduction in striping and brightness gradient compared to the "undestreaked" image.

  Subsequently, atmospheric correction was performed using ACORN (version 5, mode 1.5pb) on the destreaked 196-band image. ACORN is designed specifically for pushbroom sensors like Hyperion, which exhibit cross-track spectral calibration variation causing smile. ACORN utilizes a wavelength and full-width half-maximum (FWHM) array file generated from the center wavelength and bandwidth datasets in the HDF (L1R) file. The output of the atmospheric correction process was an atmospherically corrected image, transforming the radiance values to apparent surface reflectance.

- **Dataset 16**. The contribution of lianas to Plant Area Index (PAI) was estimated in a liana removal experiment in the secondary forest of Gigante Peninsula, Barro Colorado Nature Monument (Republic of Panama). In 2008, sixteen 80m x 80m plots were established and all trees and lianas $\geq$ 1cm DBH were measured in the central area of each plot (60m x 60m).

In 2011, immediately after a census of lianas and trees, all lianas were cut in eight randomly selected forest plots, with the remaining eight plots serving as non-manipulated control plots.

Mean per-plot Plan Area Index (PAI) were estimated as the sum of Leaf Area Index (LAI) and Wood Area Index (WAI) with the the Li-Cor LAI-2000 plant canopy analyzer (Li-Cor Biosciences, Lincoln, NE, USA). PAI measurements were taken along a grid of 49 points within the central 60 m x 60m of each plot, 50 cm and 1 m above the soil surface (n=98 measurements per plot). Simultaneously, open-sky measurements of PAI were taken every 30 seconds with a second Li-Cor LAI-2000 outside of the forest canopy on the edge of Lake Gatun. PAI measurements were restricted to the northern half of the sensor (180 degree cap) for both open-sky and within-forest measurements to ensure that the open-sky measurements did not intercept forest leaf area, and the within-forest measurements did not include the shadow of the operator. Measurements of PAI were taken fifteen days before the liana cut in 2011 in all sixteen plots, one month after the liana cut, and every consecutive year for four years. The vast majority of lianas had fallen one year after the liana cut. At every sampling period, plots were measured in the same order, ensuring consistency and comparable measurements. Plant area index (PAI) measurements were processed with the Li-Cor FV2000 Analysis Software (2005, Li-COR, Biosciences, Lincoln, NE, USA). Further details are in Rodríguez-Ronderos et al. (2016).

Calculation of f_2 using Li-Cor data. A naive but straightforward method to estimate f_2 would involve dividing the average PAI of the removal plots after liana cutting by the average PAI of the control plots, represented as (P_{rem}/P_{con}). However, this approach can introduce bias because not every tree is affected by lianas, and the extent of infestation varies among trees. Particularly, when fewer trees in a plot are infested by lianas, this estimator tends to be more biased. To address this, we derive an unbiased estimator of f_2 below.

Past research indicates that liana leaves displace tree leaves in a 1:1 ratio (Kira & Ogawa, 1971[3]). This observation is consistent with dataset 15, which revealed that the PAI post-liana removal matched the PAI in control plots four years post-cutting, as discussed in depth in Rodriguez-Ronderos et al. (2016). This consistency implies that the maximum achievable plot PAI, without lianas, closely resembles that in the control plots. Given this empirical finding, we posit that the expected maximum PAI without lianas (P_{max}) equals the observed PAI in the control plots (P_{con}).

The reduction in total PAI due to lianas is then tied to: 1) the fraction of infested trees in the plot (I), 2) the average tree infestation level (L),

[3]Kira T, Ogawa H (1971) Assessment of primary production in tropical and equatorial forests. In: Duvigneaud P (ed) Productivity of forest ecosystems. Proc Brussels Symp Unesco, Paris

and 3) the portion of liana leaves in a fully infested canopy, given as 1-f_2 when L=1. Thus, the expected PAI in a control plot is the sum of both liana and tree leaves:

$$P_{con} = I[L(1 - f_2)P_{max} + [1 - L(1 - f_2)]P_{max}] + (1 - I)[P_{max}] = P_{max} \tag{S1}$$

This equation breaks down the total PAI in a plot into contributions from infested (I) and uninfested trees (1-I). When a tree is infested, the maximum PAI is subdivided into liana and tree components using f_2 (the fraction of tree leaves) and the infestation level (L). For instance, the portion of liana leaves in an infested crown is given by $L(1 - f_2)$. As predicted, Eq. S1 simplifies to $P_{con} = P_{max}$, mirroring empirical findings. Conversely, the PAI after liana removal is expressed by:

$$\begin{aligned} P_{rem} = I[P_{max}[1 - L(1 - f_2)]] + (1 - I)[P_{max}] = \\ P_{max}[I(1 + f_2 L - L) + (1 - I)] \end{aligned} \tag{S2}$$

This doesn't simplify to P_{max}: $P_{rem} < P_{max}$ whenever $L(1 - f_2) > 0$. (i.e., when lianas add to the PAI), consistent with empirical observations. Knowing I and L, we can then solve for f_2 as:

$$f_2 = \frac{P_{max}(IL - 1) + P_{rem}}{P_{max} IL} \tag{S3}$$

Given average values of I and L for the plots (0.84 and 0.65, respectively, as per van der Heijden et al. 2015 and S. Schnitzer, personal communication), f_2 can be deduced using the observed P_{rem} - and presuming $P_{con} = P_{max}$.

- **Dataset 17**. Leaf angle data, which we obtained by collecting and analyzing leveled digital photographs, is detailed in the main text. The following figure summarizes the data distributions:

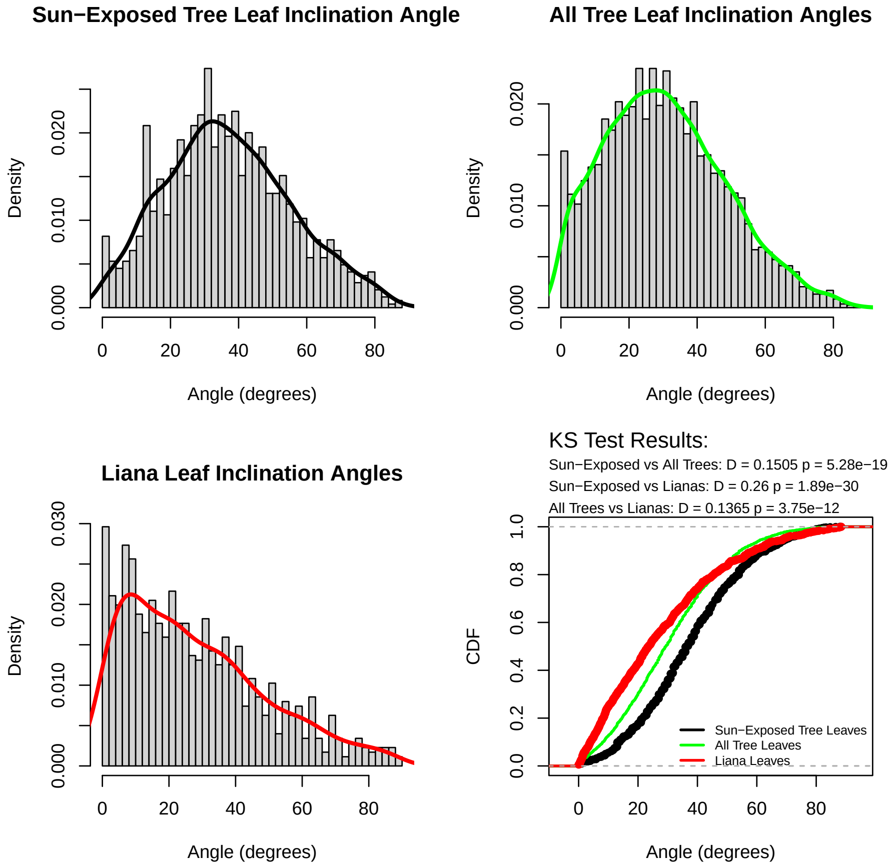

Figure S1: The distribution of leaf inclination angles for trees and lianas. The first panel (upper-left) highlights the histogram and density of tree canopy leaves (sampled at heights >20), specifically focusing on leaves in the canopy where exposure to sunlight is greatest (as virtually all liana leaves are sun-exposed). The subsequent 2 panels display the distributions for all tree leaves and liana leaves, respectively (upper-right and lower-left), followed by a combined cumulative distribution function plot (lower-right). The lower-right panel also includes results from Kolmogorov-Smirnov tests comparing these distributions. Notably, the liana leaf distribution exhibits markedly flatter angles compared to both subsets of tree leaf angles, with the greatest discrepancy observed against sun-exposed tree leaves, where there is virtually no overlap across the entire distribution.

Section S2 Bayesian model fitting

We fit leaf-level RTMs to each individual leaf spectra using the described MCMC method in the maintext and obtained RTM model parameters for each leaf (N, C_{ab}, C_{ar}, C_{br}, C_{an}, C_w, C_m). Subsequently, we employed Bayesian hierarchical models to to analyze variance across growth forms, adhering to the data's hierarchical structure: leaf measurements within species, and species within studies. Generally, the leaf-level model took the following form:

$$T_{ijk} = \beta_0 + \beta_i + \beta_{jk} + \beta_k + \epsilon \tag{S4}$$

where T_{ijk} represents one of the RTM model parameters mention above, fit for growth form i (liana or tree) from species j nested in study k. The beta parameters had normal priors with uninformative hyperpriors (following Gelman 2004[4]):

$$\beta_n \sim N(0, \sigma_n) \tag{S5}$$

where n represents one of hierachical grouping factors such as species, site or growth form.

For canopy and stand scales, we fitted the PROSAIL2 model for each site individually, without using hierarchical models to break down variance among growth forms. This is because PROSAIL2 was directly adapted to simulate liana-infested crowns/pixels as two-layer canopies with separate vertical sections (defined by f_2 and L), each characterized by growth form-specific optical parameters (see maintext). Hence, a Bayesian fit of our PROSAIL2 model already break down variance among growth forms.

We used predicted growth-form level means and variances across species and sites as prior information for leaf parameters (since the same leaf RTM is paired with the canopy-RTM). The prior possesses the mean trait value for growth-form i, allowing for variance from species, study, and other unexplained sources. This led to a largely overlapping prior distributions, with specific absolute values detailed in table S3.

We employed Markov-chain Monte Carlo (MCMC) methods to fit the model. Specifically, we established the posterior distribution for a vector of RTM parameters, denoted as a vector $\boldsymbol{\phi}$, and a vector $\mathbf{R}$ of observed reflectance values.

$$P(\boldsymbol{\phi}, \sigma \mid \mathbf{R}) \propto P(\mathbf{R} \mid \boldsymbol{\phi}, \sigma) P(\boldsymbol{\phi}) P(\sigma) \tag{S6}$$

Here P($\mathbf{R} \mid \boldsymbol{\phi}, \sigma$) is the likelihood of observing reflectance vector $\mathbf{R}$, which is assumed to be normally distributed around the simulated reflectance predicted from parameter set $\boldsymbol{\phi}$ with a variance of σ^2. P($\boldsymbol{\phi}$) and P(σ) are the prior probabilities of parameters $\boldsymbol{\phi}$ and σ.

[4]Gelman, 2004, Prior distributions for variance parameters in hierarchical models,EERI Research Paper Series No 6/2004

Each model inversion was initialized each using randomly drawn parameter values from the prior distributions (see table S2 below), and we ran the algorithm for ten independent chains until convergence. We used a burn-in of 10,000 and 100,000 iterations for leaf scale and larger scales, respectively. MCMC convergence was determined based on a value of the Gelman-Rubin multivariate potential scale reduction factor of less than 1.035 as earlier explorative analyses revealed that this cutoff improved the fit beyond the standard 1.05 cutoff (Gelman & Rubin, 1992,doi: 10.1214/ss/1177011136). Convergence typically occurred after 10,000 to 10,000,000 iterations, after burn-in, depending on model complexity. Each chain was run in parallel on a high-performance computing cluster, and we implemented a thinning interval of 10, storing only every tenth iteration, as this ensured both 1) an accurate and representative sample of the joint posterior distribution and 2) saved memory usage tenfold. After applying the burn-in and thinning filter, credible intervals (CI) were calculated as the 2.5 and 97.5 percentile of the marginal distributions of each parameter - which are estimated by sampling from the joint posterior distribution using the MCMC.

Model parameters priors in leaf level analysis (PROSPECT)

Trait	parameter	Prior	a	b
Leaf stuctural parameter	N	Uniform	1	5
Chlorophyll A & B content per unit leaf area	C_{ab}	Uniform	0	200
Carotenoid content per unit leaf area	C_{ar}	Uniform	0	50
Water mass per unit leaf area.	C_w	Uniform	0	0.1
Leaf dry matter per area	C_m	Uniform	0	0.1
Anthocyanin content	C_{an}	Uniform	0	50
brown pigments content	C_{br}	Uniform	0	10
Error	σ	Uniform	1×10^{-9}	0.1

Table S2: Prior distributions used to fit PROSPECT5,5b and D to leaf reflectance data. Columns a & b refer to the upper and lower bounds of the uniform distribution.

Stand and canopy scale model priors (PROSAIL2) for the top (1) and bottom (2) layer.

Trait	parameter	Prior	a / μ	b / σ
Leaf stuctural parameter	N_1	Normal	1.72	0.27
Chlorophyll A & B / area	$C_{ab,1}$	Normal	55.65	33.89
Carotenoid content / area	$C_{ar,1}$	Normal	10.18	4.06
Water mass / area	$C_{w,1}$	Normal	0.011	0.0056
Leaf dry matter / area	$C_{m,1}$	Normal	0.0068	0.0021
Anthocyanin content / area	$C_{an,1}$	Uniform	0	20
brown pigments content	$C_{br,1}$	Normal	0.20	0.23
Leaf stuctural parameter	N_2	Normal	1.86	0.58
Chlorophyll A & B / area	$C_{ab,2}$	Normal	65.54	32.77
Carotenoid content / area	$C_{ar,2}$	Normal	14.56	7.14
Water mass / area	$C_{w,2}$	Normal	0.019	0.012
Leaf dry matter / area	$C_{m,2}$	Normal	0.01	0.0059
Anthocyanin content	$C_{an,2}$	Uniform	0	20
brown pigments content	$C_{br,2}$	Normal	0.11	0.14
Leaf Area Index	LAI	Uniform	0	15
Leaf inclination angle	Ω	Uniform	0	90
Fraction secondary particles	f_2	Uniform	0	1
Canopy dissociation factor	D	fixed	1	1
hotspot	κ	Uniform	2.94e-05	0.04
Verticle crown cover fraction	Cv	fixed	1	NA
Crown shape	ζ	fixed	site specific	NA
Solar zenith angle	θ_s	fixed	site specific	NA
Observer zenith angle	θ_o	fixed	site specific	NA
Sun-sensor relative azimuth angle	ψ	fixed	site specific	NA
Error	σ	Uniform	1×10^{-9}	0.15

.

Table S3: Prior distributions used to fit PROSAIL2 to leaf reflectance data. Columns a / μ & b / σ refer to the upper and lower bounds of the uniform distribution or the mean and standard deviation of the normal distribution respectively. Whenever the value was fixed, the fixed value is reported in the a / μ column. Site specific parameters are taken from the scene metadata where appropriate (e.g. observer, sun and sensor angles). The prior range for the κ parameter was estimated from the min and max values calculated across all sites using published global datasets on canopy height (Healey et al. 2015, http://dx.doi.org/10.3334/ORNLDAAC/1271) and leaf sizes (Wright et al. 2017 Science, 357).

Section S3 Model robustness analyses

To get an idea of the robustness of various aspects of our inversion approach we conducted several simulation studies. Following Shiklomanov et al (2016), we identify three potential interacting error sources in our modeling study that could affect our results.

1. Model formulation error, which refers to a lack of fit, and overprediction or underprediction. We guard against model formulation error threefold. First, we evaluate the fit of each model by comparing how well the model can reproduce the observed spectral reflectance (as quantified by R^2). Second, for canopy and stand scales, we test PROSAIL2's ability to capture the difference between liana and tree signals in an unbiased fashion (e.g. on the 1:1 line). However, goodness of fit to data alone is not a sufficient test of a complex model as many free parameters can provide too much flexibility (e.g. White and Marshall 2019, doi: 10.1016/j.tree.2019.01.006). Therefore, finally, we compared whether inversely estimated differences between lianas and trees could be verified with independent measurements of the traits represented by the inverse parameters.

2. Parameter identifiability, or whether all parameters can be estimated from data. Lack of identifiability may indicate problems with the inversion procedure. We evaluated the role of parameter identifiability by simulating spectral signals from models parameterized with random parameters, and then back fit the same models using our Bayesian inversion algorithm. This analyses revealed that all free parameters were reasonably to well retrievable, i.e. with the generating parameter falling within 95% CI of the atleast 70% of the time given our prior settings.

3. Measurement error in spectra and traits (i.e. signal to noise ratios). Measurement error may systematically bias parameter estimates and lead to false differences between groups whenever groups differ in the degree of error (see e.g. Detto et al., 2019, doi: 10.1111/ele.13372). In our case, measurement error proved difficult to directly deal with because our datasets lack the repeated measurements needed to quantify measurement error. We therefore simulated the effect of increasing error in reflectance spectra to see if any differences in inversely estimated parameters could be explained by a differing error rate between groups. We found no consistent trends in how parameters responded to error in spectral data when the error rate differed among groups. Measurement error on verification traits, on the other hand, is expected to have a conservative effect on our results, i.e. it should lead to regression dilution, or a decreased ability of the model to predict physical traits from reflectance data.

Section S4 Leaf reflectance data and leaf model fits

Section S4.1 Leaf reflectance data

The figure below (Fig. S2) shows observed leaf reflectance for five plant groups, and Kullback-Leiber Divergence (KLD) which is a measure of the (theoretical) ability of classifiers to discriminate between plant groups as measured by the observed spectral distributions over wavelengths. The panel in column A, shows the measured reflectance in the solar spectrum (wavelengths between 400 to 2500 nm) of various plant species, grouped in broad categories. Here, the black dashed line indicates the mean reflectance for lianas, while the colored envelopes represent the 2.5% to 97.5% percentile interval of measured reflectance for each group. The groups are, from top to bottom, crops species, herbs and grasses, shrubs, trees and lianas (see also color legend in column C). The panel in column B, shows the distribution of KLD scores at each wavelength for each group compared to the liana distribution (bottom Panel in A). Here, KLD scores were bootstrapped at equal sample size between groups and, for lianas, within the group. The panel in column C, shows the average total divergence across all wavelengths and includes the bootstrapped 95% confidence intervals (dots and whiskers). Together the panels show that leaf reflectance compared across plants shows large variation, and substantial overlap, cautioning that dectection algorithms could confuse groups in a global analysis. In the KLD calculation, we accounted for variance sensitivity to sample size. We ensured equal sample sizes by bootstrapping at the smallest common sample size for 10,000 iterations for each group. Groups with at least 50 spectral samples were included as the expected relative error for $\sigma(\lambda)$ is then <10%. The bootstrap samples across inter- and intraspecific and site-specific variance for all plant groups. Hence, detectable significant differences imply robust distributional divergence – detectable to a classifcation algotithm without any additional information (i.e. phylogeny)

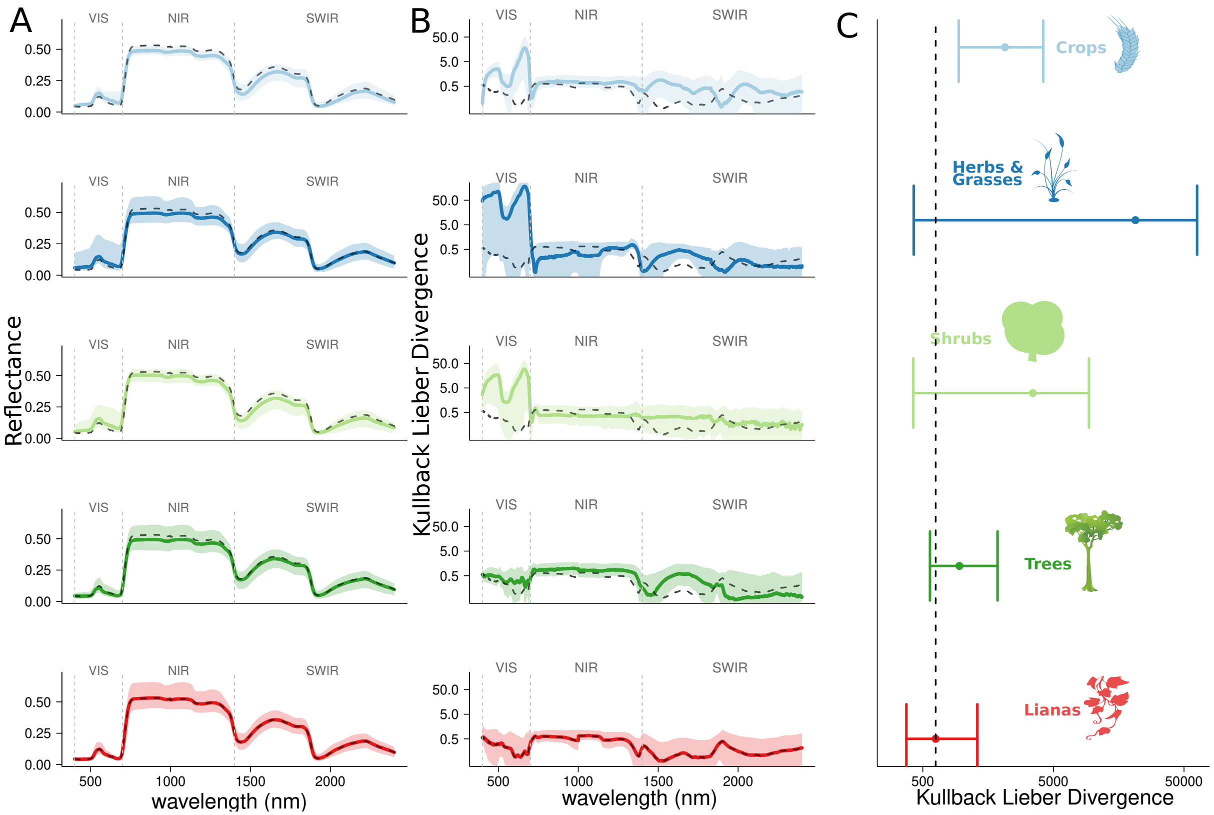

Figure S2: See above.

Section S4.2 Summary of leaf model fit statistics

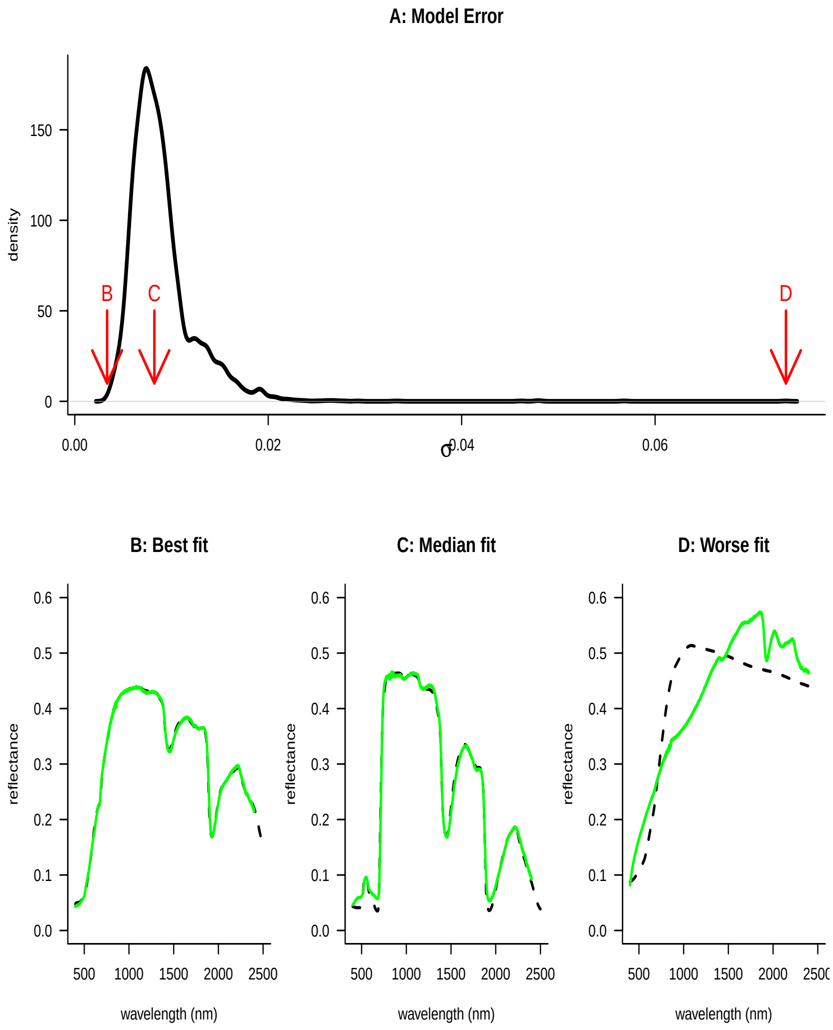

Figure S3: Leaf model fit statistics for the PROSPECTD inversion for 5424 leaves. Panel A gives the distribution of model errors (σ). In A red arrows indicate the position of the following three panels that represent the best fit (B), median fit (C) and worse fit (D). In B - D, black lines indicate the model fit, while green lines indicate the observed reflectance.

Section S4.3   Canopy model fits

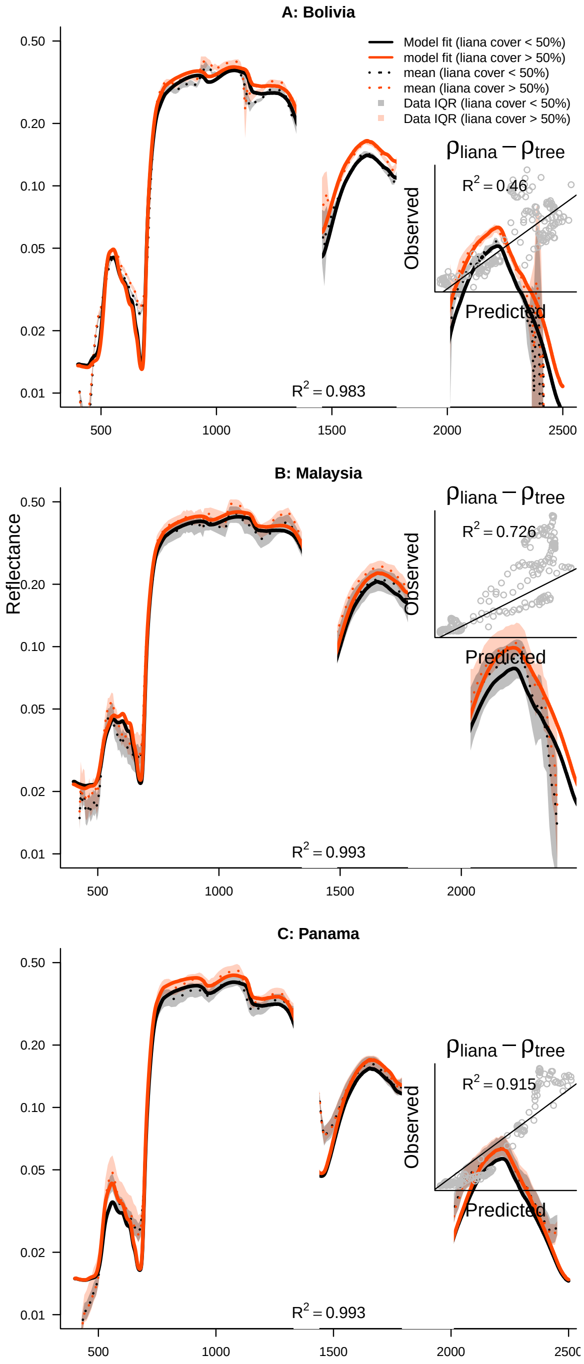

Figure S4: Alternative version of figure 4 from the maintext, with reflectance on a log-scale, so smaller reflectance values are more pronounced

Section S5 Posterior distributions

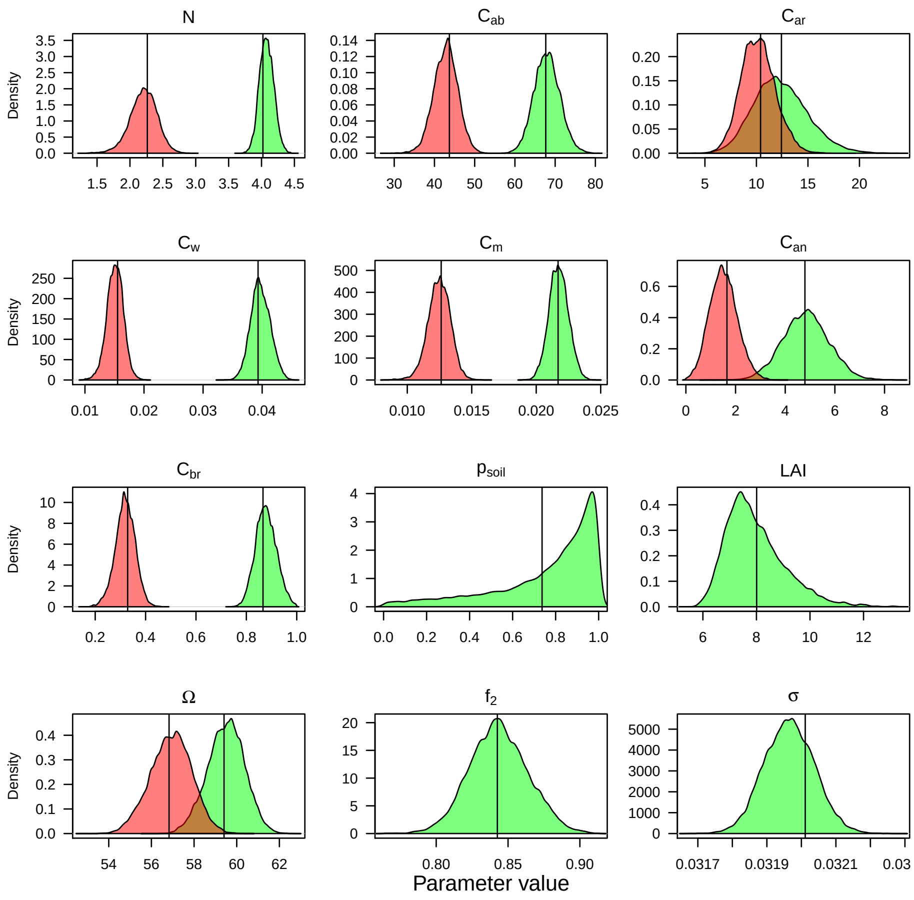

Figure S5: Marginal density plots for the posterior distributions for each model parameter of the PROSAIL2 model fit to the Bolivia dataset. Parameters that differ between lianas and trees are given in red and green respectively. Vertical lines indicate mean values for all distributions.

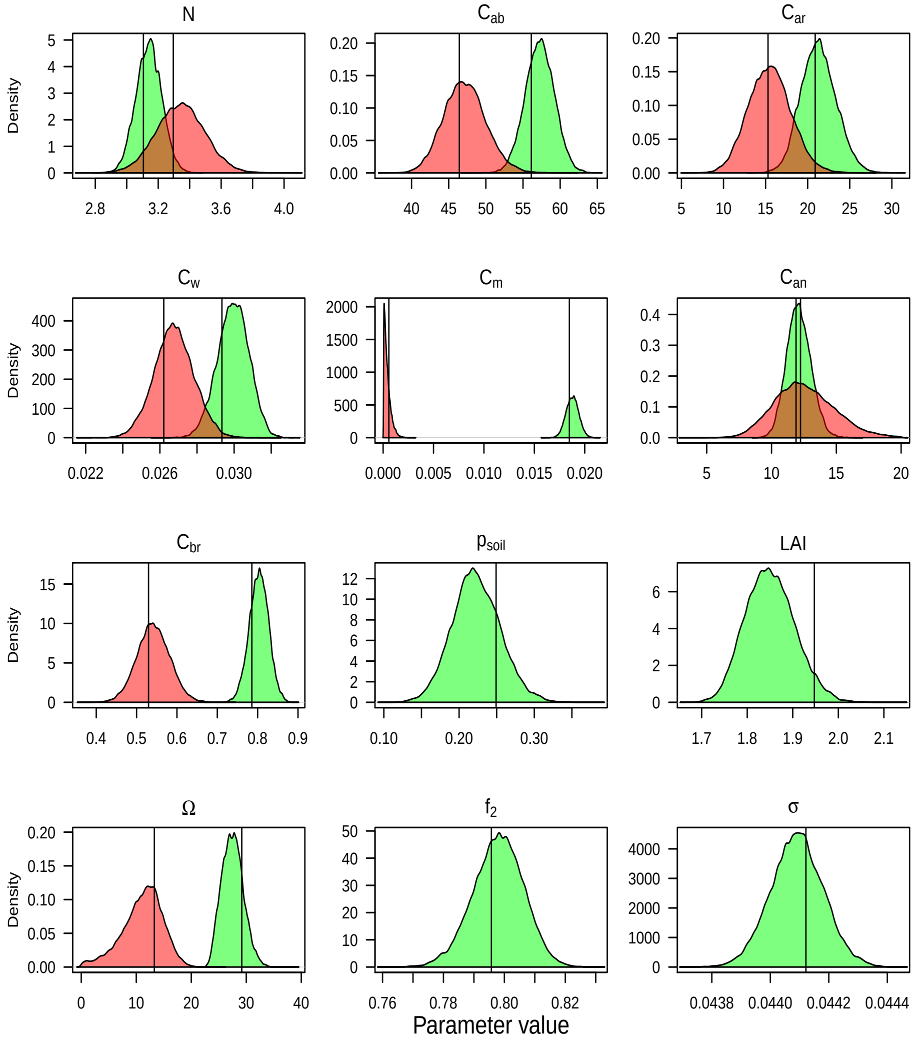

Figure S6: Marginal density plots for the posterior distributions for each model parameter of the PROSAIL2 model fit to the Malaysian dataset. Parameters that differ between lianas and trees are given in red and green respectively. Vertical lines indicate mean values for all distributions.

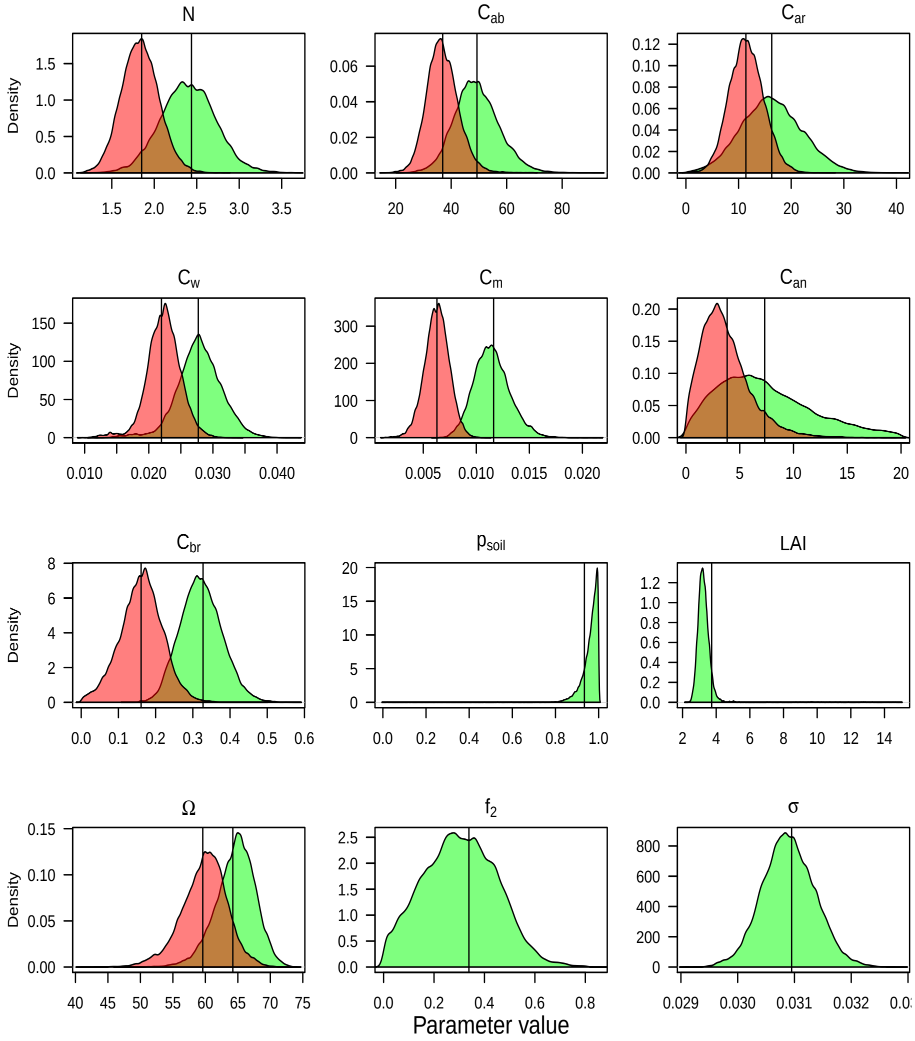

Figure S7: Marginal density plots for the posterior distributions for each model parameter of the PROSAIL2 model fit to the Panama dataset. Parameters that differ between lianas and trees are given in red and green respectively. Vertical lines indicate mean values for all distributions.

Section S6 Absolute Validation

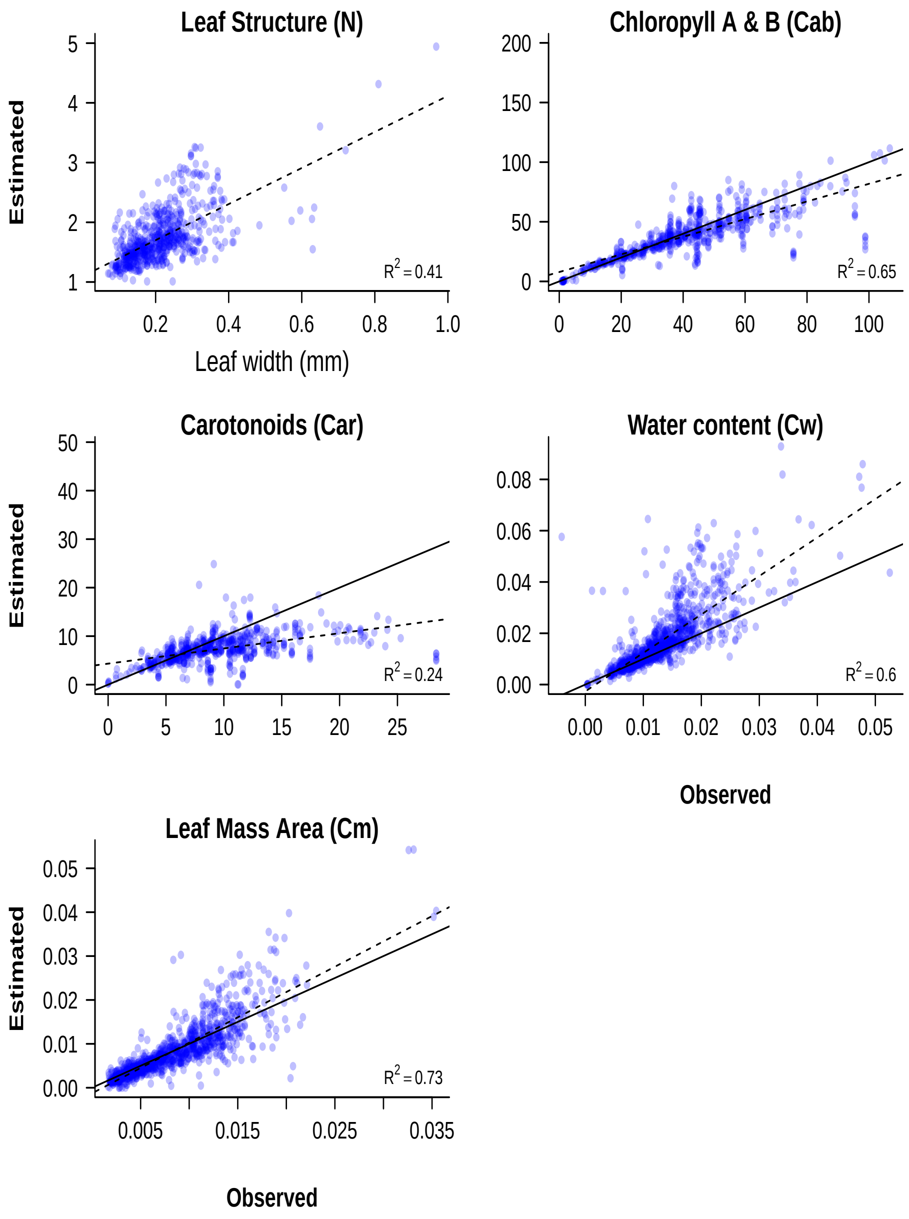

Figure S8: Leaf scale model inversion results compared to independent ground estimates for PROSPECT5B. Dashed lines show the linear relationship between x and y, as estimated by OLS. Thick black line is the 1:1 line. Note that all plots represent the same traits, and have the same units, with exception of the first panel.

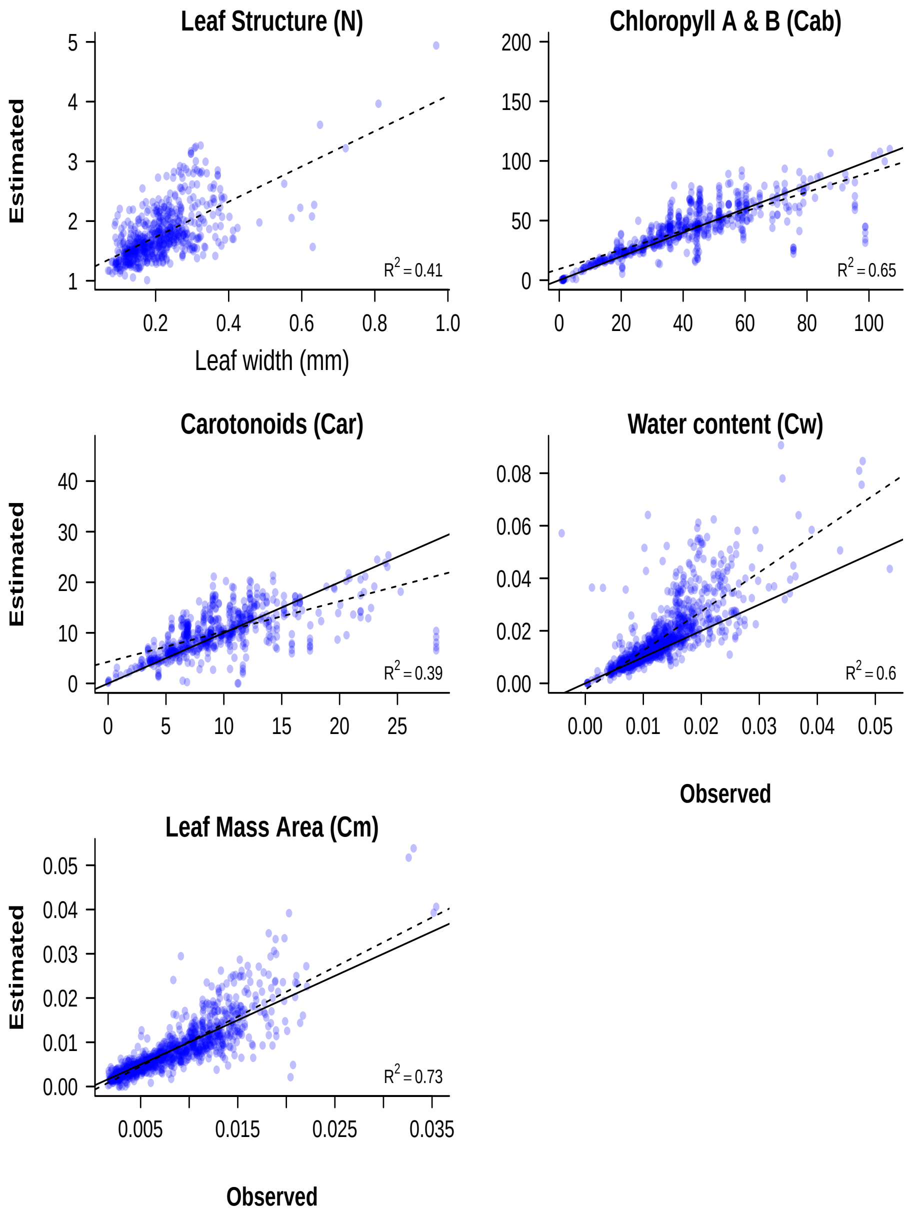

Figure S9: Leaf scale model inversion results compared to independent ground estimates for PROSPECTD. Dashed lines show the linear relationship between x and y, as estimated by OLS. Thick black line is the 1:1 line. Note that all plots represent the same traits, and have the same units, with exception of the first panel.

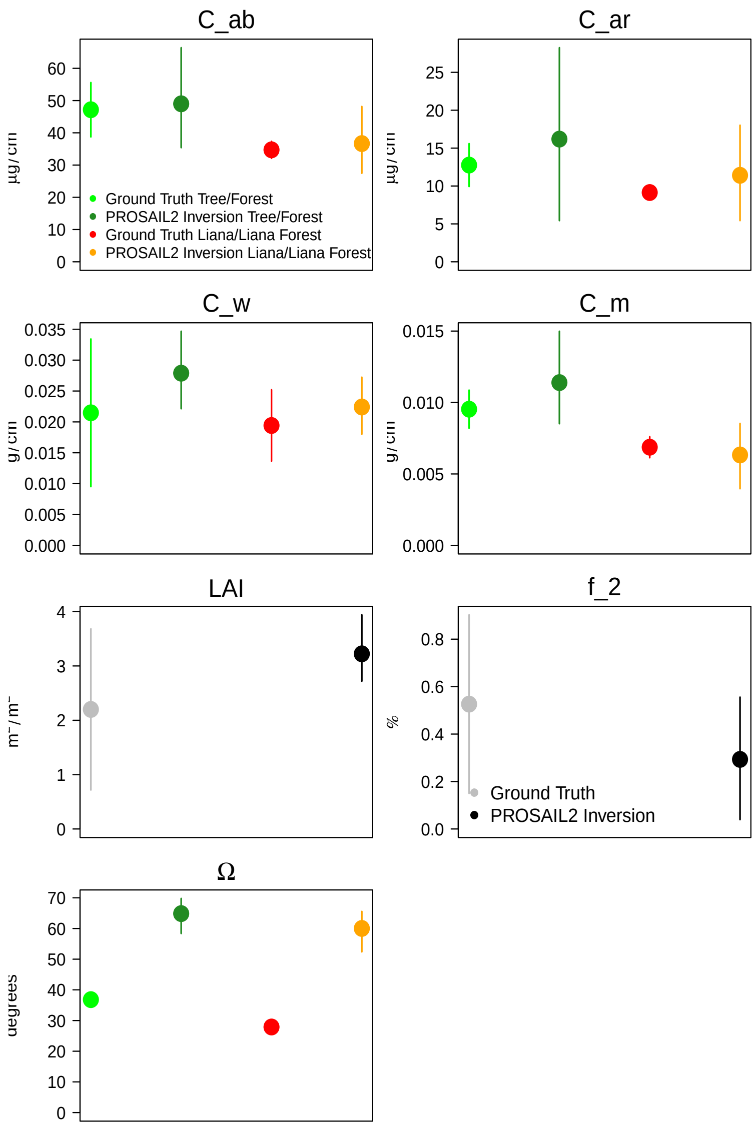

Figure S10: Canopy scale model inversion results for Panama (for Gigante) compared to independent ground estimates (from San Lorenzo, Barro Colorado Island, and PNM). Parameters that differed between lianas and trees are given in shades of red and green respectively. LAI and f_2 are given in shades of grey (see legends).

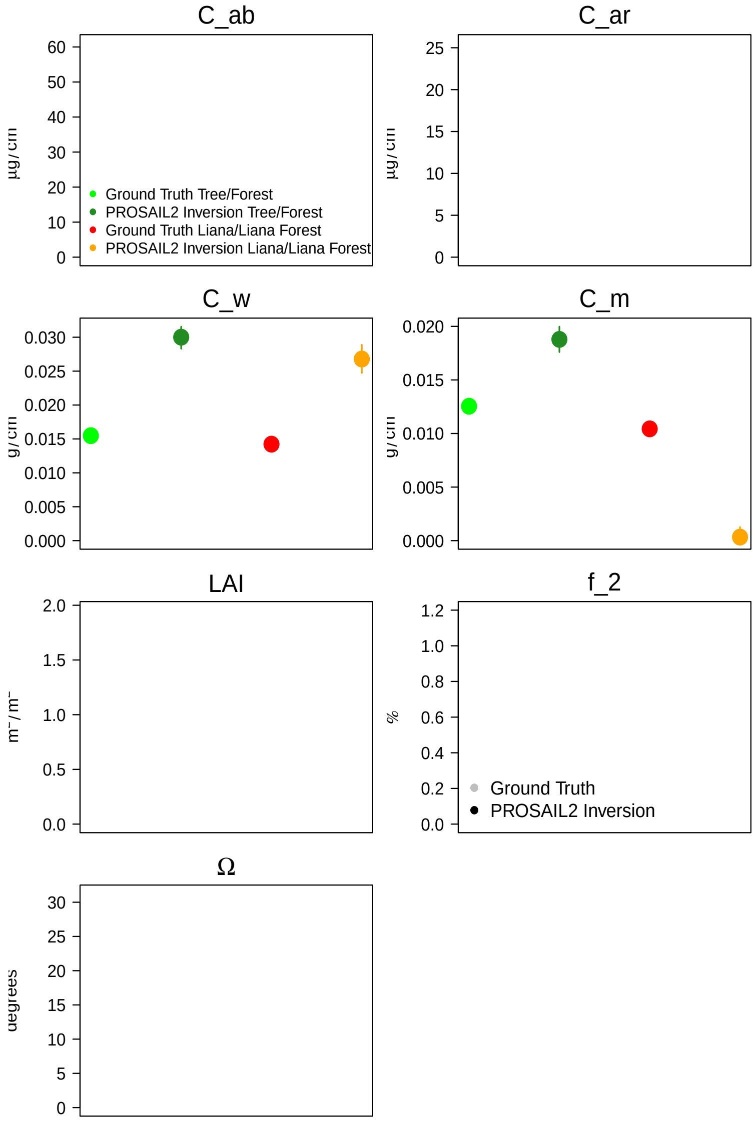

Figure S11: Canopy scale model inversion results for Malaysia (Danum Valley) compared to independent ground estimates (Sepilok). Parameters that differed between lianas and trees are given in shades of red and green respectively. LAI and f_2 are given in shades of grey (see legends). Blank plots represent traits with no ground 1validation for this study site.

Section S7 Leaf profiles

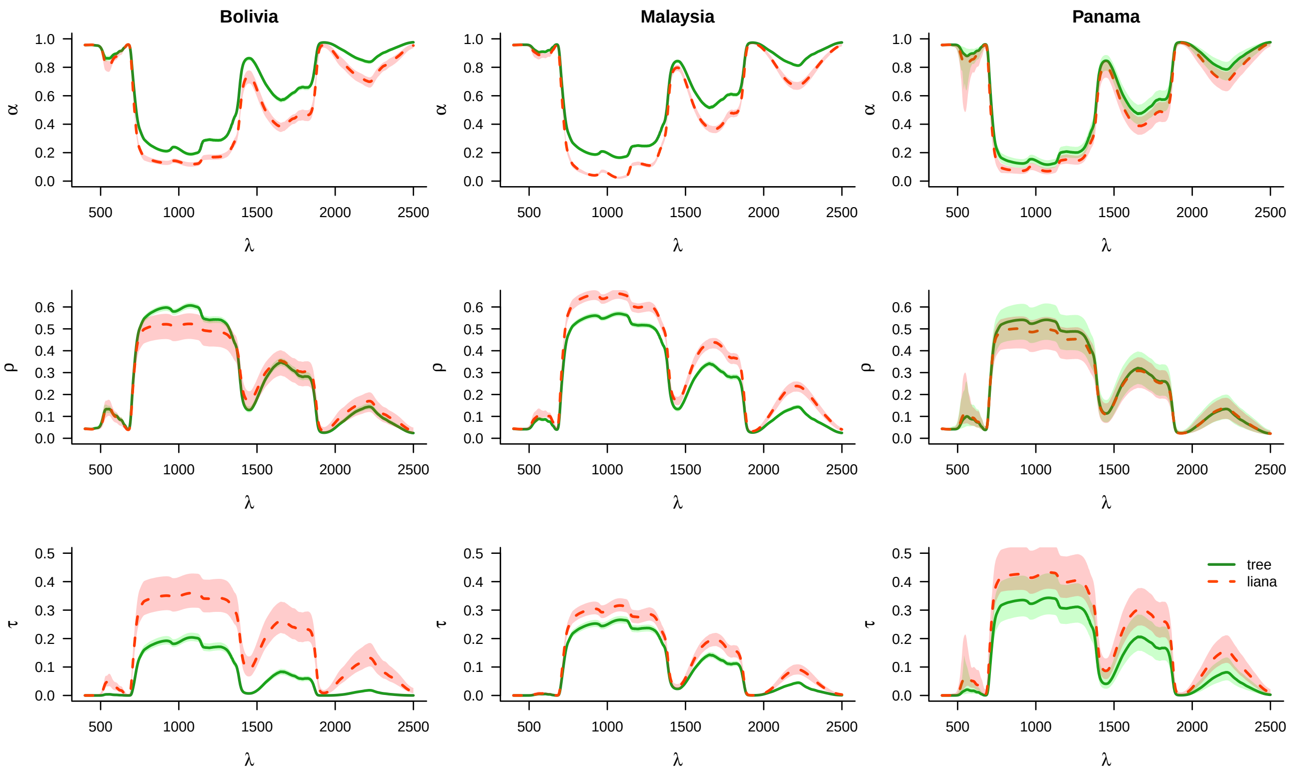

Figure S12: Leaf absorbtion (α), reflectance (ρ) and transmission (τ) profiles against wavelength (λ) for trees (green) and lianas (red) - based on the inverse PROSAIL2 fits for reflectance data collected in Bolivia, Malaysia, and Panama.

Section S8 Model experiment per site

This is the elaboration of figure 6 from the maintext towards individual sites.

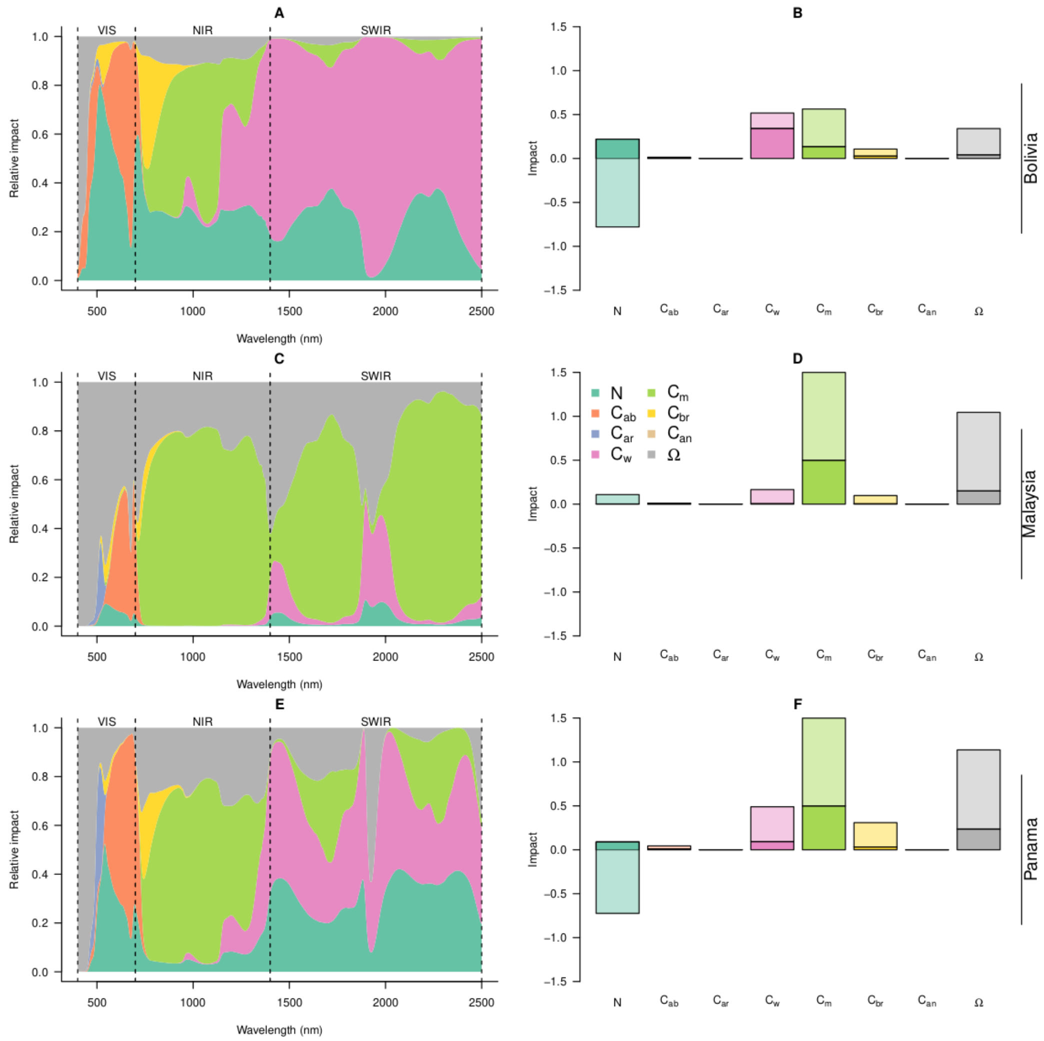

Figure S13: Model experiment showing the relative importance of different traits in generating the liana signal. In all model experiments, the traits in the top layer were set to tree traits and then iteratively replaced with liana traits either one by one (additive) or in pairs (interactive effects). At each interaction, the change in the Kullback-Leiber divergence score was calculated. Panels A, C, and E give the relative absolute impact of changing a single trait in the top canopy layer at each wavelength. Panels B, D and F give the total impact of additive (darker colors) and interactive effects (lighter colors). Here, a positive impact refers to a change in the direction of the liana signal, while a negative impact refers to in a change away from the liana signal.

Section S9 Multiple scattering

For specular flux, the energy that scatters after an interaction with a leaf particle can be roughly approximated for lianas as $(\tau_1 + \rho_1)^N$ and trees as $(\tau_2 + \rho_2)^N$ - where N gives the number of successive interactions with a leaf particles. The rate at which the transmitted energy decays (i.e. is absorbed) is then given by the derivative of the above functions: $log((\tau_1 + \rho_1))(\tau_1 + \rho_1)^N$ and $log((\tau_2 + \rho_2))(\tau_2 + \rho_2)^N$ respectively. It follows that when $(\tau_1 + \rho_1) > (\tau_2 + \rho_2)$, the energy declines at a slower rate for lianas with each interation N (as seen below in the top panel), which generally will lead to more energy being available for a secondary scattering including reflection towards the observer. The average rate of energy decline is generally much lower for lianas across the entire solar spectrum (see the bottom panel on the next page).

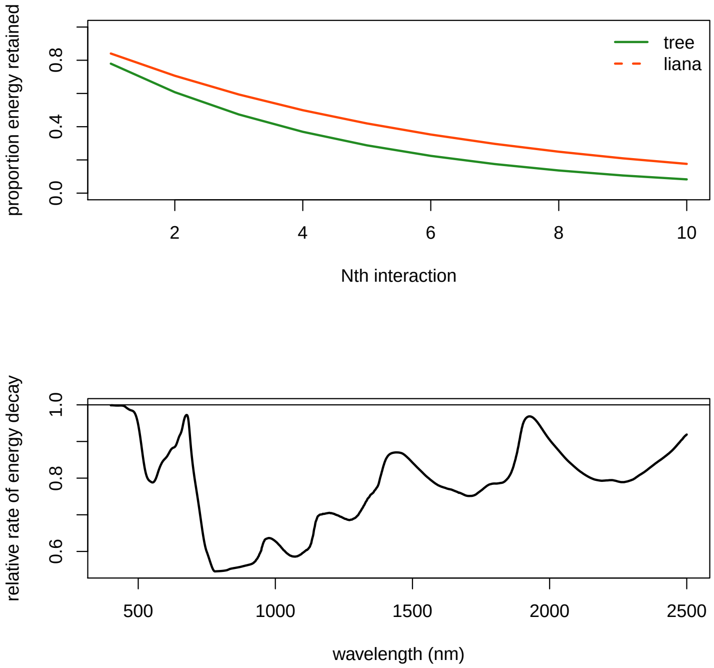

Figure S14: Top panel: The proportion of energy expected available for multiple scattering events at a wavelength of 1299 nm for trees (green) and lianas (red) based on the inverse PROSAIL2 fits for Panama. Bottom panel: The relative rate of decline for liana leaves vs tree leaves at the first interaction (N=1) - which can be up to 55% lower for lianas, a value of 1 means that energy declines equally for interactions with liana vs tree leaves, a value < 1 incates it declines slower for lianas.

Section S10 References

Asner, G. P., D. E. Knapp, J. Boardman, R. O. Green, T. Kennedy-Bowdoin, M. Eastwood, R. E. Martin, C. Anderson, and C. B. Field. 2012. Carnegie Airborne Observatory-2: Increasing science data dimensionality via high-fidelity multi-sensor fusion. *Remote Sensing of Environment* 124:454–465. https://doi.org/10.1016/j.rse.2012.06.012

Chandler, C. J., G. M. F. van der Heijden, D. S. Boyd, C. E. Waite, M. E. J. Cutler, H. Costa, R. Nilus, and G. M. Foody. 2021. Remote sensing liana infestation in an aseasonal tropical forest: addressing mismatch in spatial units of analyses. *Remote Sensing in Ecology and Conservation*.

Chavana-Bryant, C., Y. Malhi, J. Wu, G. P. Asner, A. Anastasiou, B. J. Enquist, E. G. Cosio Caravasi, C. E. Doughty, S. R. Saleska, R. E. Martin, and F. F. Gerard. 2017. Leaf aging of Amazonian canopy trees as revealed by spectral and physiochemical measurements. *New Phytologist* 214:1049–1063. https://doi.org/10.1111/nph.13853

Datt, B., T. R. McVicar, T. G. Van Niel, D. L. B. Jupp, and J. S. Pearlman. 2003. Preprocessing EO-1 Hyperion hyperspectral data to support the application of agricultural indexes. *IEEE Transactions on Geoscience and Remote Sensing* 41:1246–1259. https://doi.org/10.1109/TGRS.2003.813206

Detto, M., M. D. Visser, S. J. Wright, and S. W. Pacala. 2019. Bias in the detection of negative density dependence in plant communities. *Ecology Letters* 22:1923–1939. https://doi.org/10.1111/ele.13372

Foster, J. R., P. A. Townsend, and C. E. Zganjar. 2008. Spatial and temporal patterns of gap dominance by low-canopy lianas detected using EO-1 Hyperion and Landsat Thematic Mapper. *Remote Sensing of Environment* 112:2104–2117.

Gelman, A. 2004. Prior distributions for variance parameters in hierarchical models. EERI Research Paper Series No. 6/2004.

Gelman, A., and D. B. Rubin. 1992. Inference from iterative simulation using multiple sequences. *Statistical Science* 7:457–472. https://doi.org/10.1214/ss/1177011136

Goodenough, D. G., et al. 2003. Processing Hyperion and ALI for forest classification. *IEEE Transactions on Geoscience and Remote Sensing* 41:1321–1331. https://doi.org/10.1109/TGRS.2003.813214

Healey, S. P., M. W. Hernandez, D. P. Edwards, M. A. Lefsky, E. Freeman, P. L. Patterson, E. J. Lindquist, and A. J. Lister. 2015. CMS: GLAS LiDAR-derived Global Estimates of Forest Canopy Height, 2004–2008. ORNL DAAC, Oak Ridge, Tennessee, USA. https://doi.org/10.3334/ORNLDAAC/1271

Hosgood, B., S. Jacquemoud, G. Andreoli, V. Verdebout, G. Pedrini, and I. Schmuck. 1994. Leaf Optical Properties EXperiment 93 (LOPEX93). Report EUR 16095 EN. European Commission, Joint Research Centre, Institute for Remote Sensing Applications, Ispra, Italy.

Kira, T., and H. Ogawa. 1971. Assessment of primary production in tropical and equatorial forests. Pages 309–321 in P. Duvigneaud, editor. *Productivity*

of forest ecosystems. Proceedings of the Brussels Symposium, UNESCO, Paris, France.

Meireles, J. E. 2020. Spectral dataset. figshare. https://doi.org/10.6084/m9.figshare.12449147.v1

NERC Airborne Research and Survey Facility, D. F. R. P. Burslem, and D. Coomes. 2019. ARSF 2014_309 – MA14_14 flight: Airborne remote sensing measurements. Centre for Environmental Data Analysis, 25 September 2019. https://doi.org/10.5285/c708cad9950c45b1af0d4e9ca944f09a

Nunes, M. H., S. Both, B. Bongalov, C. Brelsford, S. Khoury, D. F. R. P. Burslem, C. Philipson, N. Majalap, T. Riutta, and D. A. Coomes. 2019. Changes in leaf functional traits of rainforest canopy trees associated with an El Niño event in Borneo. *Environmental Research Letters* 14:085005. https://doi.org/10.1088/1748-9326/ab2eae

Pavan, G., S. Jacquemoud, G. D. Rosny, J. P. Rambaut, J. Frangi, L. P. R. Bidel, C. François, and D. J. Mulla. 2004. RAMIS: A new portable field radiometer to estimate leaf biochemical content.

Rodríguez-Ronderos, M. E., et al. 2016. Effects of liana removal on the water relations of host trees in a Panamanian tropical forest. *Ecology* 97:3271–3277.

Sánchez-Azofeifa, A. 2025. Replication data for: When can we detect lianas from space? Towards a mechanistic understanding of liana-infested forest optics. Harvard Dataverse. https://doi.org/10.7910/DVN/OG95TV

Sánchez-Azofeifa, G. A., K. Castro, S. J. Wright, J. Gamon, M. Kalacska, B. Rivard, S. A. Schnitzer, and J. L. Feng. 2009. Differences in leaf traits, leaf internal structure, and spectral reflectance between two communities of lianas and trees: Implications for remote sensing in tropical environments. *Remote Sensing of Environment* 113:2076–2088.

Serbin, S. P., J. Wu, K. S. Ely, E. L. Kruger, P. A. Townsend, R. Meng, B. T. Wolfe, A. Chlus, Z. Wang, and A. Rogers. 2019. From the Arctic to the tropics: Multibiome prediction of leaf mass per area using leaf reflectance. *New Phytologist* 224:1557–1568. https://doi.org/10.1111/nph.16123

Shiklomanov, A. N., M. C. Dietze, T. Viskari, P. A. Townsend, and S. P. Serbin. 2016. Quantifying the influences of spectral resolution on uncertainty in leaf trait estimates through a Bayesian approach to RTM inversion. *Remote Sensing of Environment* 183:226–238.

Tymen, B., M. Réjou-Méchain, J. W. Dalling, S. Fauset, T. R. Feldpausch, N. Norden, O. L. Phillips, B. L. Turner, J. J. Viers, J. J. Chave. 2016. Evidence for arrested succession in a liana-infested Amazonian forest. *Journal of Ecology* 104:149–159.

Tymen, B., M. Réjou-Méchain, J. W. Dalling, et al. 2019. Data from: Evidence for arrested succession in a liana-infested Amazonian forest. Dryad. https://doi.org/10.5061/dryad.1pc19

U.S. Geological Survey. 2019. EO-1 Hyperion data. U.S. Geological Survey, Earth Resources Observation and Science (EROS) Center, Sioux Falls, South Dakota, USA. https://doi.org/10.5066/P9JXHMO2. Image ID: EO1H2300702001248112PP_SGS_01 (acquired 2001-09-05), available via https://earthexplorer.usgs.gov/

van der Heijden, G. M. F., J. S. Powers, and S. A. Schnitzer. 2015. Lianas reduce carbon accumulation and storage in tropical forests. *Proceedings of the National Academy of Sciences USA* 112:13267–13271. https://doi.org/10.1073/pnas.1504869112

Visser, M., M. Detto, F. Meunier, et al. 2025. Data from: When can we detect lianas from space? Towards a mechanistic understanding of liana-infested forest optics. Dryad. https://doi.org/10.5061/dryad.xpnvx0krs

Wright, I. J., et al. 2017. Global climatic drivers of leaf size. *Science* 357:917–921. https://doi.org/10.1126/science.aal4760

Wu, J., C. Chavana-Bryant, N. Prohaska, S. P. Serbin, K. Guan, L. P. Albert, X. Yang, W. J. D. van Leeuwen, A. J. Garnello, G. Martins, Y. Malhi, F. Gerard, R. C. Oliviera, and S. R. Saleska. 2017. Convergence in relationships between leaf traits, spectra and age across diverse canopy environments and two contrasting tropical forests. *New Phytologist* 214:1033–1048. https://doi.org/10.1111/nph.14051

Wu, J., H. Kobayashi, S. C. Stark, R. Meng, K. Guan, N. N. Tran, S. Gao, W. Yang, N. Restrepo-Coupe, T. Miura, R. C. Oliviera, A. Rogers, D. G. Dye, B. W. Nelson, S. P. Serbin, A. R. Huete, and S. R. Saleska. 2018. Biological processes dominate seasonality of remotely sensed canopy greenness in an Amazon evergreen forest. *New Phytologist* 217:1507–1520.

Wu, J., N. Prohaska, and S. Saleska. 2012. Leaf reflectance spectra of tropical trees in Tapajos National Forest. EcoSIS Spectral Library. http://ecosis.org
